# TrkB signaling regulates the cold-shock protein RBM3-mediated neuroprotection

**DOI:** 10.26508/lsa.202000884

**Published:** 2021-02-09

**Authors:** Diego Peretti, Heather L Smith, Nicholas Verity, Ibrahim Humoud, Lis de Weerd, Dean P Swinden, Joseph Hayes, Giovanna R Mallucci

**Affiliations:** 1 UK Dementia Research Institute at the University of Cambridge and Department of Clinical Neurosciences, Island Research Building, Cambridge Biomedical Campus, Cambridge, UK; 2 MRC Toxicology Unit at the University of Cambridge, Leicester, UK

## Abstract

Increasing levels of the cold-shock protein, RNA-binding motif 3 (RBM3), either through cooling or by ectopic over-expression, prevents synapse and neuronal loss in mouse models of neurodegeneration. To exploit this process therapeutically requires an understanding of mechanisms controlling cold-induced RBM3 expression. Here, we show that cooling increases RBM3 through activation of TrkB via PLCγ1 and pCREB signaling. RBM3, in turn, has a hitherto unrecognized negative feedback on TrkB-induced ERK activation through induction of its specific phosphatase, DUSP6. Thus, RBM3 mediates structural plasticity through a distinct, non-canonical activation of TrkB signaling, which is abolished in RBM3-null neurons. Both genetic reduction and pharmacological antagonism of TrkB and its downstream mediators abrogate cooling-induced RBM3 induction and prevent structural plasticity, whereas TrkB inhibition similarly prevents RBM3 induction and the neuroprotective effects of cooling in prion-diseased mice. Conversely, TrkB agonism induces RBM3 without cooling, preventing synapse loss and neurodegeneration. TrkB signaling is, therefore, necessary for the induction of RBM3 and related neuroprotective effects and provides a target by which RBM3-mediated synapse-regenerative therapies in neurodegenerative disorders can be used therapeutically without the need for inducing hypothermia.

## Abstract (precis)

The neuroprotective effects of the cold-shock protein RBM3 have broad appeal for therapy. RBM3 induction through cooling occurs through a non-canonical pattern of TrkB signaling. The pathway can now be targeted pharmacologically for RBM3-mediated neuroprotection without the need for cooling.

## Introduction

In the healthy adult brain, synapses are continually lost and replaced through structural plasticity, a process critical for repair that also underlies learning and memory (Kandel et al, 2014; Bailey et al, 2015). Failure of this process leads to synapse loss and, eventually, neuronal demise. Structural plasticity also occurs as an adaptive response in hypothermia, including the dismantling and reassembly of synapses on cooling and rewarming, respectively (Von Der Ohe et al, 2006, 2007; Popov et al, 2007; Horowitz & Horwitz, 2019). We previously showed that cold-induced structural plasticity is mediated by the cold-shock protein, RNA binding motif 3 (RBM3), which is necessary for synapse number maintenance in healthy wild-type mice. Furthermore, we showed that failure to induce RBM3 expression underlies the impaired synapse regenerative capacity underlying the earliest stages of synapse loss in several mouse models of neurodegenerative disease (Peretti et al, 2015). Critically, increasing neuronal RBM3 levels, induced either through cooling or by lentivirally-mediated overexpression, restores synapse number and is profoundly neuroprotective in prion-diseased and in Alzheimer’s mouse models, rescuing cognitive deficits, preventing neuronal loss, and markedly increasing survival (Peretti et al, 2015) RBM3’s neuroprotective effects appear to be mediated largely through its downstream effector, reticulon 3 protein, RTN3 (Bastide et al, 2017), although the exact mechanism of synapse regeneration is unknown. Others have shown that RBM3 mediates the protective effects of hypothermia in brain slices (Chip et al, 2011). Furthermore, RBM3 is known to increase global protein synthesis rates (Dresios et al, 2004), particularly in dendrites (Smart et al, 2007) and to regulate neuronal polarity (Pilotte et al, 2018) and neurogenesis during development (Xia et al, 2018), which may contribute to its effects on structural plasticity.

Thus, the therapeutic induction of RBM3 is an appealing novel target for neurodegenerative diseases. Therapeutic hypothermia is widely used—and is highly effective—in the treatment of various ischemic and traumatic brain conditions in humans and also for neuroprotection, through mechanisms that are unclear (Yenari & Han, 2012; Karnatovskaia et al, 2014). RBM3 is expressed in human cells and brain tissue and increases on cooling (Danno et al, 1997; Archer et al, 2014; Jackson et al, 2018) and has been proposed as a biomarker in therapeutic hypothermia (Rosenthal et al, 2019). Inducing RBM3 expression pharmacologically represents a potential novel means of neuroprotection, in the absence of hypothermia. This requires an understanding of the mechanisms of RBM3 induction, which also bring new mechanistic insights into the biology of hypothermia.

Using our established cooling-rewarming paradigm for induction of RBM3 and structural plasticity in wild-type mice (Peretti et al, 2015; Bastide et al, 2017), we show that RBM3 expression is induced through brain-derived neurotrophic factor (BDNF)-TrkB signaling involving activation of the PLCγ1-CREB branch on cooling leading to a non-canonical inhibition of p-ERK branch activation by RBM3 through a previously unknown feedback loop. This pattern of TrkB activation appears necessary for the coordination of structural plasticity, where RBM3-mediated inhibition of p-ERK underlies synapse dismantling on cooling before reassembly on rewarming. This capacity is lost in RBM3-null neurons. We find that both genetic reduction in TrkB levels in vivo and pharmacological antagonism of TrkB signaling in vivo and in vitro abrogate RBM3 induction on cooling and abolish RBM3-mediated structural plasticity. Furthermore, we provide proof-of-principle that pharmacological induction of the cold-shock proteins RBM3 and RTN3 can be achieved, independent of hypothermia. Finally, we show that the marked neuroprotective effects of cooling in prion-diseased mice are abolished by pharmacological inhibition of the pathway by preventing RBM3 induction, highlighting its relevance as a novel target for therapeutic modulation for neurodegenerative diseases. Thus, TrkB signaling is necessary and sufficient for the induction of RBM3 and its related neuroprotective effects and, critically, provides a means for its therapeutic activation without the need for cooling.

## Results

### Cooling induces a non-canonical pattern of TrkB signaling associated with RBM3 induction in vivo

**Figure 1.**
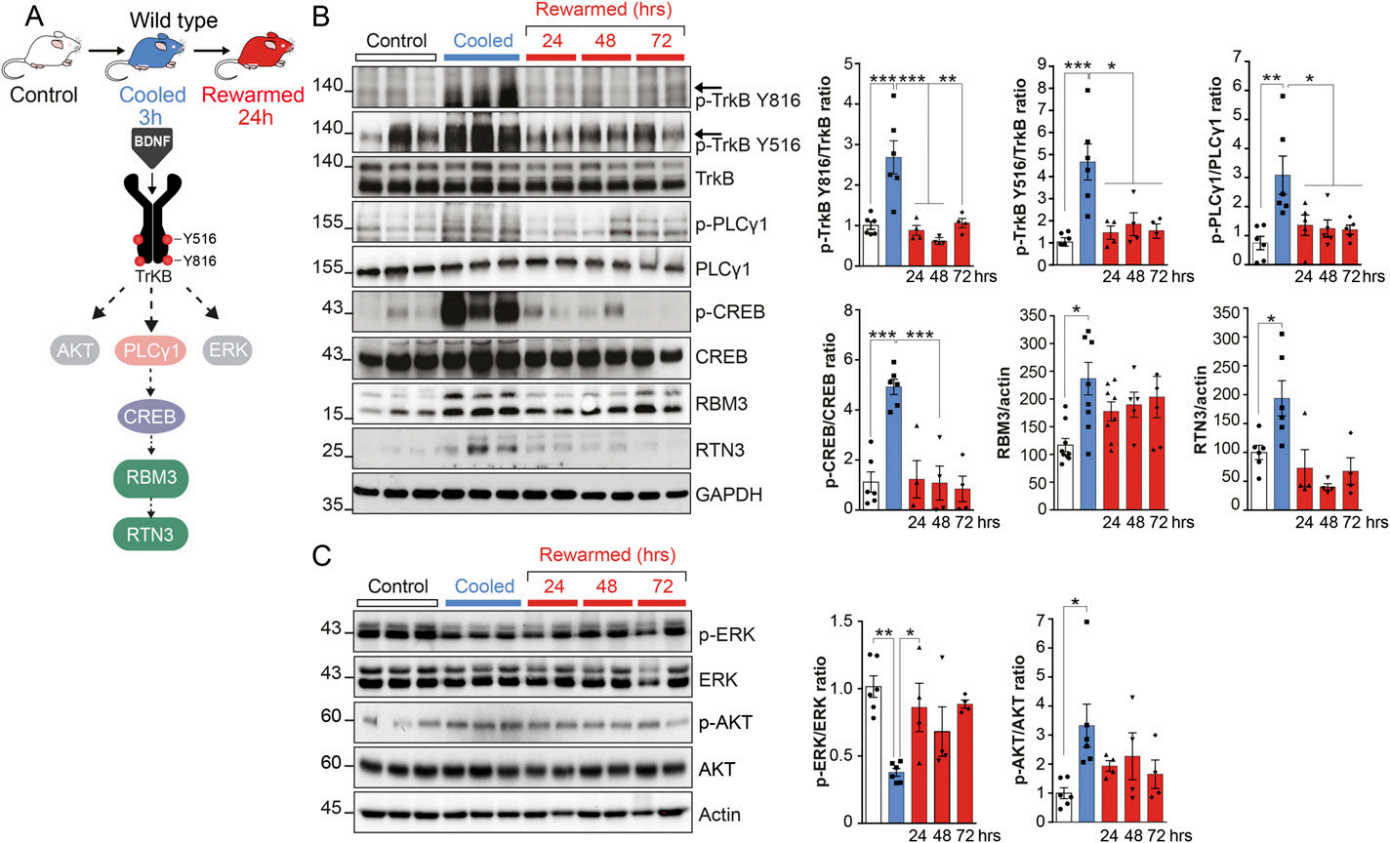
Cooling induces a non-canonical pattern of TrkB signaling associated with RBM3 induction in vivo. **(A)** Schematic showing cooling/rewarming sequence in wild-type mice and TrkB signaling on cooling leading to RBM3 and RTN3 expression. **(B)** Western blot analysis of TrkB signaling shows activation of PLCγ1-CREB pathway in control, cooled and rewarmed mice. TrkB Tyr 516 (*P* = 0.0004); TrkB Tyr 816 (*P* = 0.0003); CREB Ser 133 (*P* < 0.0001); RBM3 (*P* = 0.0101); RTN3 (*P* = 0.0405). **(C)** Western blots showing TrkB activation of AKT and ERK branches. Cooling reduced p-ERK and increased p-AKT on cooling. AKT (*P* = 0.0278); ERK (*P* = 0.0011). **(B, C)** Representative Western blots of (B) and (C) belong to the same set of experiments with loading controls GAPDH or Actin used for quantification. Bar charts show mean ± SEM. *P < 0.05, **P < 0.01, ***P < 0.001, one-way ANOVA and Tukey’s multiple comparisons test. For all experiments, n = 4–6 mice per condition.

**Figure S1.**
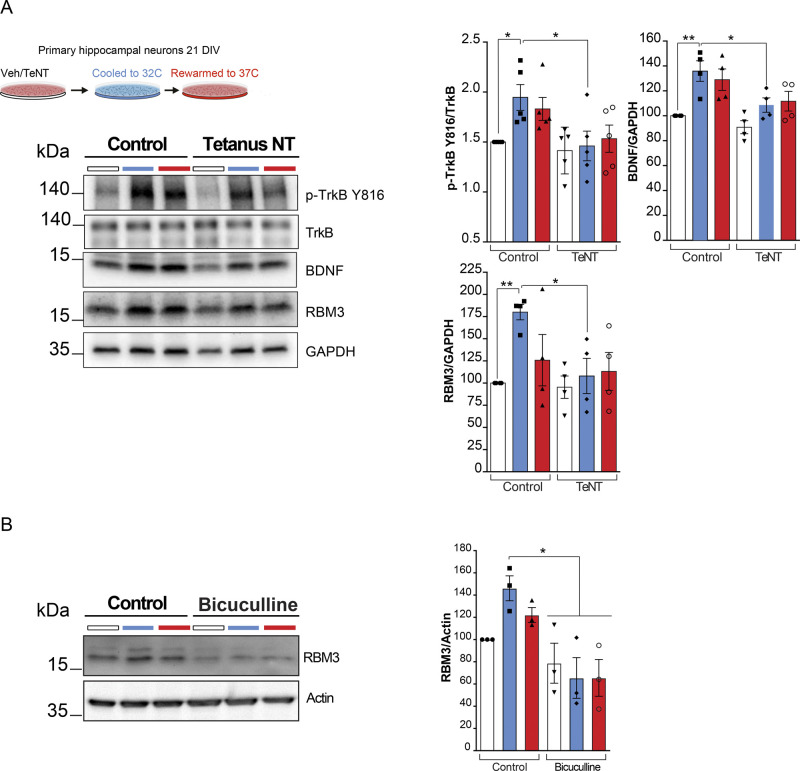
BDNF increases during cooling and is blocked by tetanus neurotoxin. **(A)** Primary hippocampal neurons were cultured for 19–20 d and subjected to cooling at 32°C for 24 h before rewarming. Blocking TrkB activation using the tetanus neurotoxin prevented BDNF increase. Two-way ANOVA test and Tukey’s multiple comparisons test, *P < 0.05, **P < 0.01, TrkB Tyr 816 control + vehicle versus cooled + vehicle *P* = 0.0478; cooled + vehicle versus cooled + TeNT (*P* = 0.0265); BDNF control + vehicle versus cooled + vehicle (*P* = 0.0062); cooled + vehicle versus cooled + TeNT (*P* = 0.0451); RBM3 cooled + vehicle versus cooled + TeNT (*P* = 0.0060); cooled + vehicle versus cooled + TeNT (*P* = 0.0139). n = 4 cultures per condition. **(B)** Primary hippocampal neurons were cultured for 19–20 d and subjected to cooling at 32°C for 24 h before rewarming. Blocking with GABA_A_ receptor antagonist bicuculline (40 μM) prevented RBM3 induction. Vehicle control versus bicuculline control (*P* = 0.0389), bicuculline cooled (*P* = 0.0121), and bicuculline rewarmed (*P* = 0.0121). N = 3 cultures per condition.

We have previously described the induction of RBM3 on cooling and defined its role in structural plasticity in wild-type mice (Peretti et al, 2015; Bastide et al, 2017). Thus, we used wild-type mice to determine the mechanism controlling RBM3 expression. Both human and mouse RBM3 have predicted binding sequences in the promoter for the transcription factor CREB (CREB target gene database http://natural.salk.edu/CREB/). We, therefore, examined BDNF-TrkB signaling, which plays a major role in synapse plasticity through p-CREB induction (Poo, 2001; Benito & Barco, 2010; Cheng et al, 2011; Camandola & Mattson, 2017) as a potential route of RBM3 induction (Fig 1A). On binding BDNF, the TrkB receptor is activated by autophosphorylation on specific tyrosine residues, conserved between mice and humans, which regulate signaling through various downstream cascades. Phosphorylation at tyrosine (Tyr) 516 is involved in signal transduction events including MAPK/ERK and PI3K/Akt pathways; Tyr 706/707 enhances kinase activity and Tyr 816 recruits PLCγ1 (Segal et al, 1996) and ultimately p-CREB (Huang & Reichardt, 2003; Minichiello, 2009). We first confirmed that cooling hippocampal neurons in vitro induces BDNF release, which is abolished by tetanus toxin, which blocks pre-synaptic BDNF release (Fig S1A). Furthermore, as BDNF is involved in activity-dependent synaptic scaling (Rutherford et al, 1998), we tested the induction of RBM3 levels on cooling with bicuculline (40 mM), which induces synaptic downscaling (Schanzenbächer et al, 2016). Bicuculline abrogated RBM3 induction, supporting its induction being, at least in part, activity dependent (Fig S1B).

We next measured TrkB signaling in C57/Bl6J mice at the time-points that we have previously examined for structural plasticity: 2–3 h post-cooling to 18–20°C and 24 h after rewarming, which produce robust dismantling and reassembly of synapses, respectively (Peretti et al, 2015; Bastide et al, 2017). We found that cooling in vivo significantly increased TrkB receptor phosphorylation at residues Tyr 516, Tyr 706/707 (not shown), and Tyr 816 in hippocampi 2 h post-cooling, followed by return to near-basal levels upon rewarming after 24 h (Fig 1B). Activation of PLCγ1 by phosphorylation at Tyr 783, also increased on cooling, accompanied by a highly significant increase in phosphorylation of CREB at Ser133, both of which returned to baseline levels on rewarming (Fig 1B). As expected, the cold-shock proteins RBM3 and RTN3 were both markedly elevated (Fig 1B), with RBM3 levels remaining elevated for at least 72 h, consistent with previous reports (Peretti et al, 2015). We also examined other major branches of TrkB signaling and found activation of Akt at Ser 473 (Fig 1C), consistent with cold-induced p-TrkB Tyr 516 activation. However, in contrast to canonical BDNF-TrkB signaling, we found that cooling resulted in reduction in phosphorylation of ERK1/2, followed by recovery upon rewarming (Fig 1C). Thus, cooling induces a non-canonical pattern of TrkB signaling associated with RBM3 induction, characterized by activation of the PLCγ1 branch, but with inhibition of, rather than activation, of p-ERK signaling.

**Figure S2.**
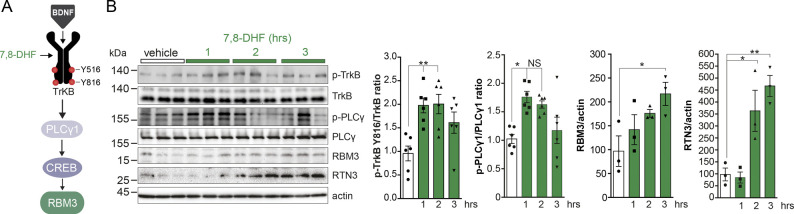
Specific TrkB agonist 7,8-DHF induces the cold-shock proteins RBM3 and RTN3 independent of cooling in vivo. **(A)** Schematic showing the cold-induced BDNF-TrkB pathway and intervention with the TrkB agonist 7,8-DHF. **(B)** Western blots showing activation of TrkB signaling in mice after treatment with the TrkB agonist 7,8-DHF (5 mg/kg). Quantification of Western blots (right). All bar charts show mean ± SEM. One-way ANOVA and Tukey’s multiple comparisons test, *P < 0.05, **P < 0.01, N.S., not significant, TrkB Tyr 816 control + vehicle versus 7,8-DHF, 1 h and control + vehicle versus 7,8-DHF, 2 h (*P* = 0.0055 and *P* = 0.0043); RBM3 control + vehicle versus 7,8-DHF, 3 h (*P* = 0.0443); RTN3 control + vehicle versus 7,8-DHF, 2 h and control + vehicle versus 7,8-DHF, 3 h (*P* = 0.0269 and *P* = 0.0042); Kruskal–Wallis test and Dunn’s multiple comparisons test, Tyr 783 PLCγ1 control + vehicle versus 7,8-DHF, 1 h and control + vehicle versus 7,8-DHF, 2 h (*P* = 0.0066 and *P* = 0.0341). n = 6 mice per time point.

Furthermore, consistent with this signaling pathway, mice treated with the specific TrkB agonist 7,8-dihydroxyflavone (7,8-DHF) (Fig S2A) (Jang et al, 2010; Liu et al, 2014) in the absence of cooling showed elevation of both RBM3 and RTN3 proteins in parallel with TrkB phosphorylation at Tyr 816 and PLCγ1 activation (Fig S2B), providing proof-of-principle that pharmacological induction of cold-shock proteins can be achieved independently of hypothermia.

### Genetic and pharmacological inhibition of TrkB signaling prevents RBM3 induction in vivo

**Figure 2.**
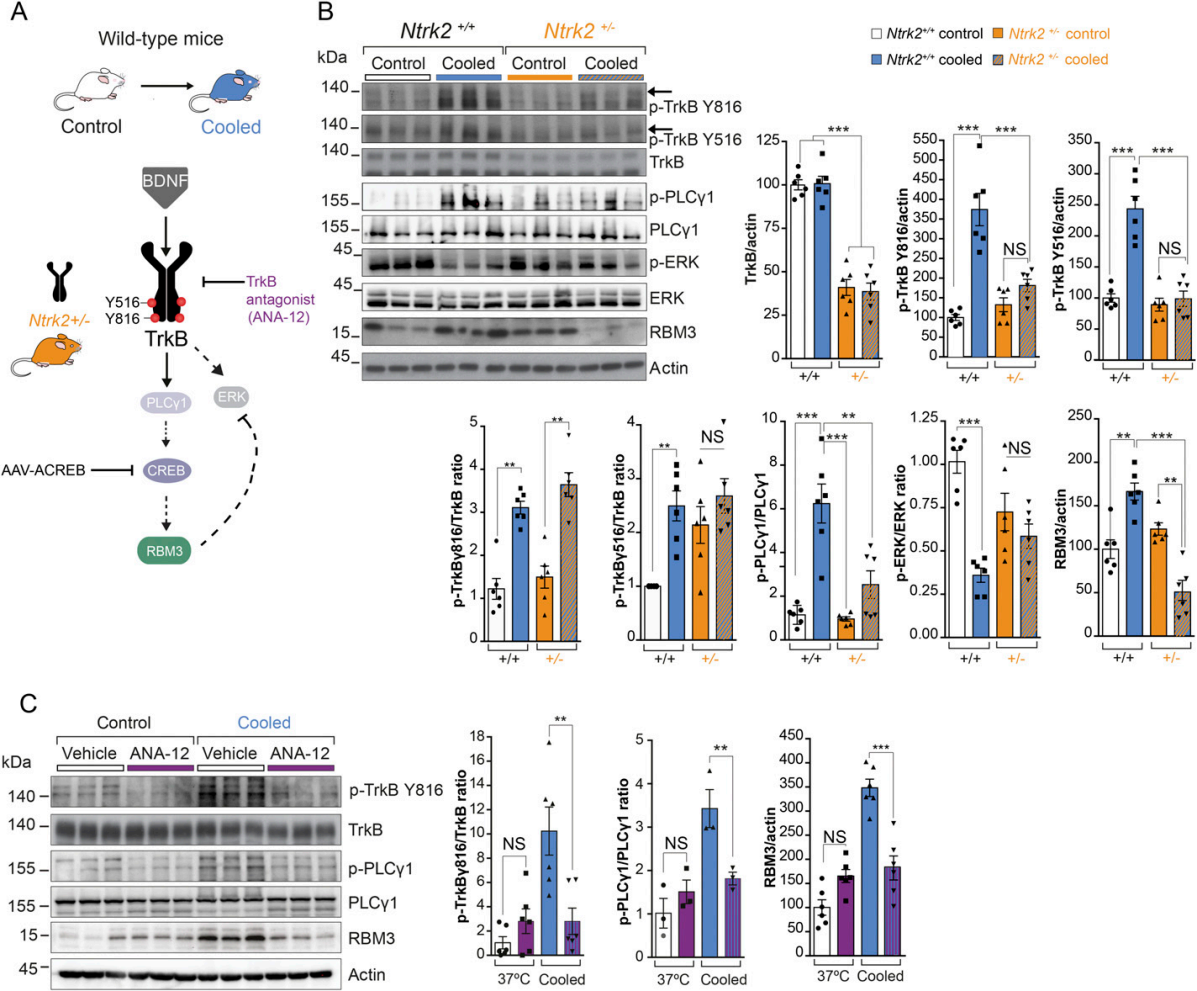
Genetic and pharmacological inhibition of TrkB signaling prevents RBM3 induction on cooling in vivo. **(A)** Schematic showing the in vivo modulation of TrkB signaling on cooling: genetic modulation is via Ntrk2^+/−^mice that are hemizygous for TrkB receptor and ACREB; pharmacological antagonism is via ANA-12. **(B)** TrkB haploinsufficiency abrogated cold-dependent RBM3 induction with a failure to activate the pattern of TrkB signaling seen in control mice with normal TrkB expression. Western blots and bar charts showing markers of the TrkB pathway in Ntrk2^+/+^ and Ntrk2^+/−^ samples pre and post cooling. RBM3 (*P* < 0.0001). TrkB Tyr 516; TrkB Tyr 816 are shown as total normalized directly to actin or as relative levels normalized to the basal level of TrkB receptor. **(C)** TrkB antagonist ANA-12 prevented RBM3 induction on cooling. One-way ANOVA, and Tukey’s multiple comparisons test. *P < 0.05, **P < 0.01, ***P < 0.001, N.S., not significant, TrkB Tyr 816 (*P* = 0.0008); PLCγ1 Tyr 783 (*P* = 0.0007); RBM3 (*P* = 0.0074). Bar charts show mean ± SEM. For all experiments, n = 6 mice per time point. Source data are available for this figure.

**Figure S3.**
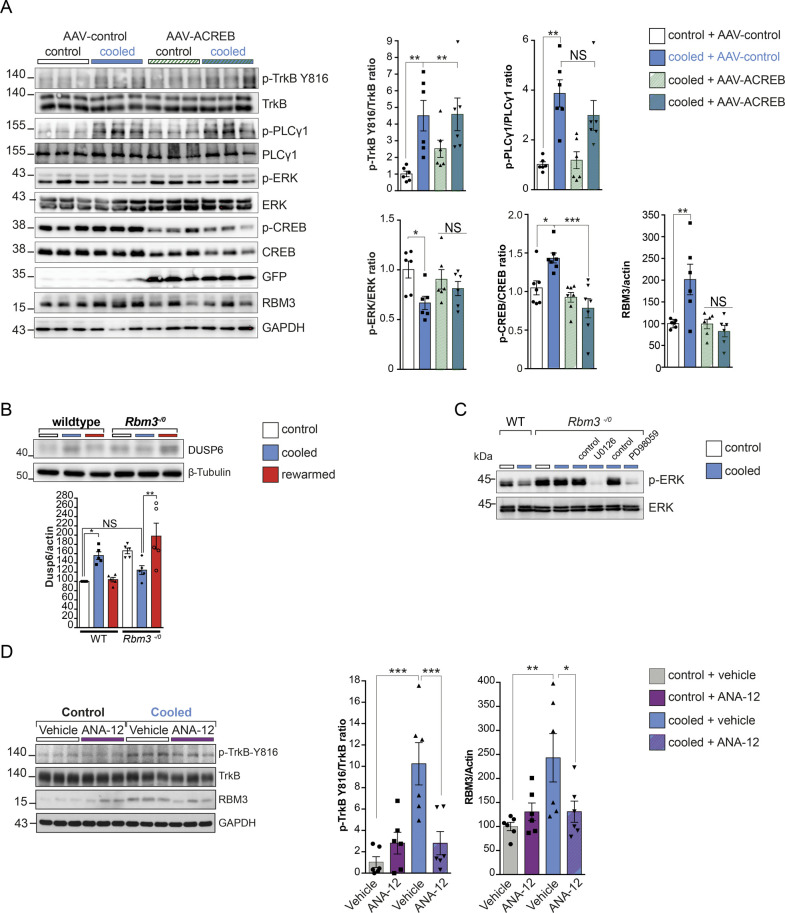
Modulation of TrkB signaling at various levels. **(A)** Dominant negative inhibitor ACREB prevents CREB phosphorylation and RBM3 induction on cooling. Infection with adeno-associated virus (AAV)-GFP-T2A-ACREB prevented the induction of RBM3 on cooling in vivo. Upstream TrkB pathway is activated independent of CREB inhibition but p-ERK inhibition is prevented. Quantification of Western blots (right). All bar charts show mean ± SEM. One-way ANOVA and Tukey’s multiple comparisons test, *P < 0.05, **P < 0.01, ***P < 0.001, N.S., not significant, TrkB Tyr 816 (AAV-empty control versus AAV-empty cooled [*P* = 0.0028]; AAV-empty control versus AAV-ACREB cool [*P* = 0.0023]); Tyr 783 PLCγ1 (AAV-empty control versus AAV-empty cooled [*P* = 0.0035]); Thr202/Thr204 ERK (AAV-empty control versus AAV-empty cooled [*P* = 0.0343]); S133 CREB (AAV-empty control versus AAV-empty cooled [*P* = 0.0382], AAV-empty cooled versus AAV-ACREB cooled [*P* = 0.0005]); RBM3 (AAV-empty control versus AAV-empty cooled [*P* = 0.0073]). n = 6 mice per condition. **(B)** RBM3 controls the induction of the ERK-specific phosphatase Dusp6 on cooling in vitro in Rbm3^−/0^ neurons. Western blot shows Dusp6 increase on cooling of wild-type neurons. Rbm3^−/0^ neurons showed no increase in Dusp6 on cooling. Bar chart shows quantification of Western blots (right); mean ± SEM. Two-way ANOVA and Tukey’s multiple comparisons test, *P < 0.05, **P < 0.01, N.S., not significant, Dusp6 wild-type control versus wild-type cooled (*P* = 0.0299); Rbm3^−/0^ cooled versus Rbm3^−/0^ rewarmed (*P* = 0.0028). n = 5 cultures per condition. **(C)** ERK inhibitors U0126 and PD98059 reduce ERK phosphorylation on cooling in vitro in Rbm3^−/0^ neurons. Representative Western blots showing the effect of U0126 and PD98059 inhibitors on ERK Thr202/Thr204 phosphorylation. Wild-type and Rbm3^−/0^ neurons at 37°C (white bar), or 32°C (blue bar) untreated, or treated with vehicle or the respective ERK inhibitors U0126 and PD98059. **(D)** ANA-12 antagonist prevents TrkB-mediated RBM3 induction in prion diseased mice. Representative Western blots showing inhibition of TrkB phosphorylation (Y816) and induction of RBM3 mediated by ANA-12 TRkB antagonist. Bar charts quantifications. Two-way ANOVA and Tukey’s multiple comparisons test, *P < 0.05, **P < 0.01, ***P < 0.001, N.S., not significant, TrkB Tyr 816 control + vehicle versus vehicle cooled + vehicle (*P* = 0.0003), cooled + vehicle versus cooled + ANA-12 (*P* = 0.0025); RBM3 (control + vehicle versus cooled + vehicle [*P* = 0.0020], cooled + vehicle versus cooled + ANA-12 [*P* = 0.0137]). n = 6 mice per condition. Source data are available for this figure.

To test whether TrkB-PLCγ1 signaling is necessary for cooling-induced RBM3 expression in vivo, rather than simply being an associated finding, we manipulated the pathway genetically and pharmacologically at various levels (Fig 2A). First, we tested the capacity for RBM3 expression on cooling in mice hemizygous for the TrkB gene (Ntrk2^+/−^). These animals express ∼40% total TrkB receptor levels as assessed by Western blotting (Fig 2B) compared with control (non-hemizygous Ntrk2^+/+^) littermates and have a predominantly C57Bl/6J genetic background, similar to the wild-type mice tested above. On cooling, Ntrk2^+/−^ mice showed increased phosphorylation of TrkB receptor at tyrosine residue 816, similar to wild-type mice, but not at residue 516 (Fig 2B: using pTrkB/TrkB ratios, hence allowing for reduced levels of the receptor); although when total levels of pTrkB were quantitated with respect to actin, TrkB activation was significantly reduced compared with those in wild-type mice. However, overall, Ntrk2^+/−^ mice showed reduced downstream signaling at the level of p-PLCγ1 levels and total failure of RBM3 induction on cooling (Fig 2B), supporting reduced signaling through the pathway in TrkB hemizygous mice. Furthermore, the reduction in p-ERK levels seen on cooling in wild-type mice (Fig 1C) was abolished in Ntrk2^+/−^ mice (Fig 2B). Thus, there is a TrkB gene-dosage effect that supports a direct role for this pathway in cold-induced RBM3 expression. Consistent with this, the specific small molecule TrkB antagonist, ANA-12 (Cazorla et al, 2011) significantly reduced cooling-induced TrkB phosphorylation at Tyr 816, PLCγ1 activation and RBM3 induction (Fig 2C). Thus, both genetic and pharmacological reduction of TrkB signaling at the level of the receptor blocked downstream RBM3 induction on cooling. Focal expression of ACREB, a dominant-negative form of CREB, via adeno-associated virus (AAV) injection into the hippocampus also blocked RBM3 expression in contrast to control virus. Critically, upstream pathway activation was unchanged by ACREB expression but cooling-induced p-ERK inhibition was absent (Fig S3A), suggesting that RBM3 itself inhibits ERK activation through a feedback loop (see below and Fig 4). Collectively, the results support the conclusion that RBM3 induction on cooling is mediated by p-TrkB-p-PLCγ1-p-CREB signaling with subsequent RBM3-mediated inhibition of p-ERK.

### Blocking TrkB pathway activation abolishes cooling-induced structural plasticity in wild-type neurons

**Figure 3.**
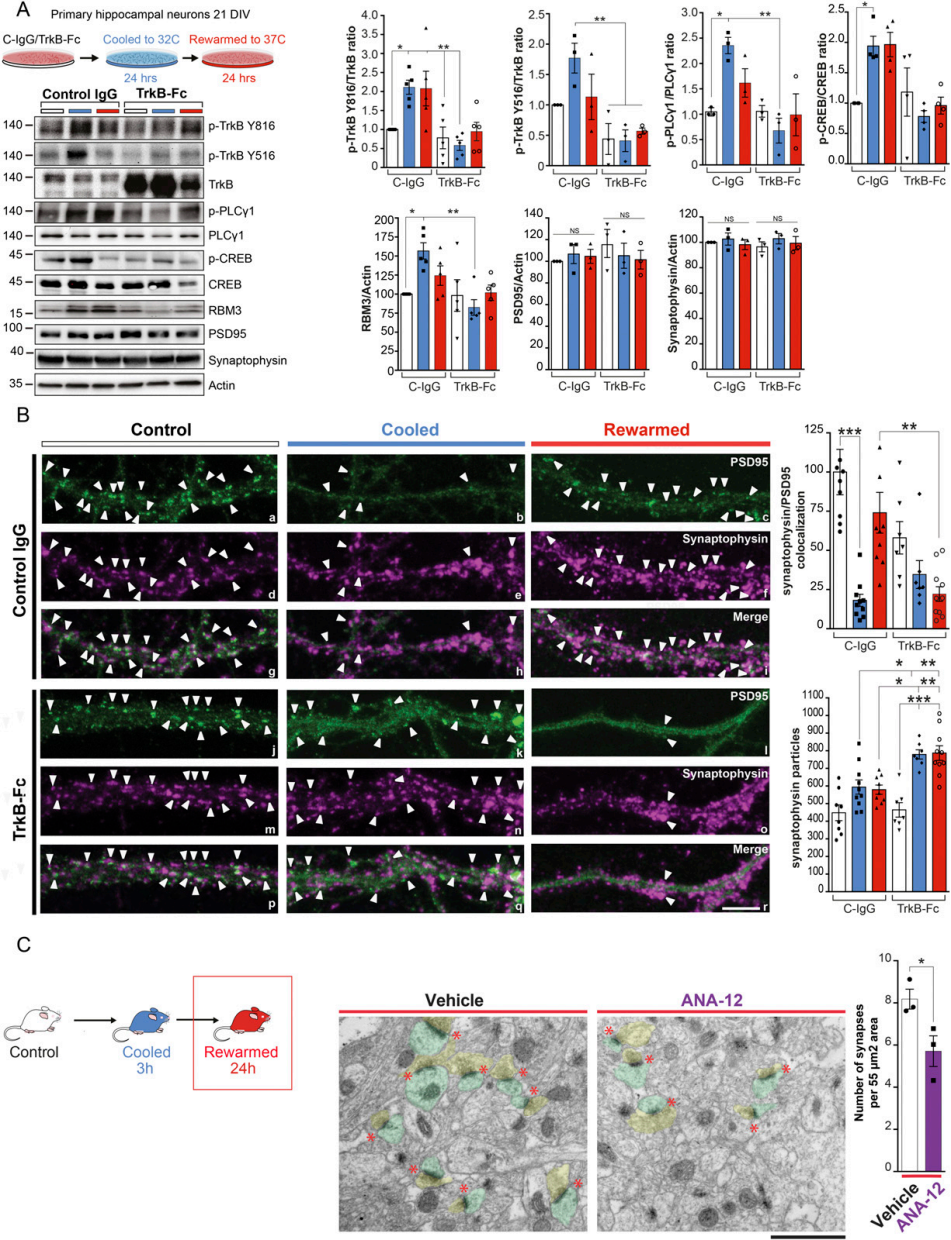
Blocking TrkB activation abolishes cooling-induced structural plasticity in wild-type neurons. **(A)** Primary hippocampal neurons were cultured for 19–20 d and subjected to cooling at 32°C for 24 h before re-warming. Blocking TrkB activation using the ligand scavenger TrkB-Fc prevented TrkB-PLCγ1-CREB signaling and RBM3 induction on cooling. Control IgG treated neurons showed pathway activation as wild-type mice (Fig 1B). Total levels of synaptic protein PSD95 are unchanged. Bar charts show mean ± SEM. Two-way ANOVA and Tukey’s multiple comparisons test, *P < 0.05, **P < 0.01, TrkB Tyr 816 (*P* = 0.0014); Tyr 516 (*P* = 0.0025); Tyr 783 PLCγ1 (*P* = 0.0043); CREB Ser 133 (*P* = 0.0218); RBM3 (*P* = 0.0036). n = 3–5 cultures per condition. **(B)** TrkB-Fc prevented synapse recovery following cooling. Confocal images of representative dendritic fragments labeled for the presynaptic protein synaptophysin (magenta) and the post-synaptic protein PSD95 (green). White arrowheads point toward synapses. Top panels show control IgG-treated, bottom panels show TrkB-Fc treated samples. Bar chart shows quantification of co-localized synaptophysin and PSD95 puncta (right). One-way ANOVA, *P < 0.05, **P < 0.01, (C-IgG control versus C-IgG cooled < 0.0001; C-IgG cooled versus C-IgG rewarmed *P* = 0.0014; C-IgG rewarmed versus TrkB-Fc rewarmed *P* = 0.0027). Bar chart shows numbers of synaptophysin particles. One-way ANOVA, *P < 0.05, **P < 0.01, ***P < 0.001, (C-IgG cooled versus TrkB-Fc cooled *P* = 0.0182; C-IgG cooled versus TrkB-Fc rewarmed *P* = 0.0044; C-IgG rewarmed versus TrkB-Fc cooled *P* = 0.0109; C-IgG rewarmed versus TrkB-Fc rewarmed *P* = 0.0025; TrkB-Fc control versus TrkB-Fc cooled *P* < 0.0001). n = 3 cultures per condition. Scale bar, 10 μm. **(C)** The TrkB-specific antagonist ANA-12 impairs synapse recovery on rewarming after cooling in wild-type mice. Representative EM micrographs and graph bar are shown. Red stars indicate synapses; which have been pseudo-colored pre- (yellow) and post- (green) synaptic compartments. *P < 0.05, *t* test (vehicle versus ANA-12 *P* = 0.0466). n = 3 mice per condition.

Cooling-induced structural plasticity involves two phases: first, synapse disassembly, involving dispersal of the post-synaptic density and reduction in synapse number and second, regeneration or reassembly of synapses on rewarming, with recovery in numbers (Popov & Bocharova, 1992). We previously showed that RBM3 is needed for this process to occur in vivo (Peretti et al, 2015). We now asked whether TrkB activation mediates RBM3-dependent structural plasticity in primary hippocampal neuronal cultures. We first confirmed that the same signaling pathway occurs in vitro (Fig 3A) as observed in vivo (Fig 1B). To induce RBM3 expression, neurons were cooled to 32°C for 24 h and then rewarmed to 37°C for a further 24 h, as described (Knight et al, 2015, 2016; Bastide et al, 2017). Cells were treated with TrkB-Fc, a scavenger of the TrkB ligand, BDNF (Chen et al, 2010; Edelmann et al, 2014) and control cultures with IgG alone. Control cultures showed the described pattern of TrkB pathway activation on cooling (Fig 1B). This was blocked by TrkB-Fc treatment (Fig 3A), with reduced levels of pTrkB 816 and 516, p-PLCγ1 and pCREB and failure of RBM3 induction (Fig 3A), consistent with the effect of ANA-12 in vivo (Fig 2C). We next tested the effects on structural plasticity. TrkB-Fc abolished structural plasticity and prevented recovery on rewarming seen in control neurons (Fig 3B, panels j–r; arrowheads mark synapses at points of co-localization; and bar graph). The effect was particularly marked on the pattern of PSD95 labeling on re-warming (Fig 3B, compare panels c and l), although total levels of PSD95 (and of synaptophysin) remained unchanged (Fig 3A) and total number of pre-synaptic synaptophysin particles are increased in TrkB-Fc treated neurons. It is likely that cooling and rewarming differentially affect pre-synaptic markers, but overall, fewer synapses are present in cooled conditions and their numbers increase on rewarming. Importantly, this effect depends on TrkB as it is blocked by TrkB-Fc. Total synapse numbers in TrkB-Fc-treated neurons were also lower at baseline than in control IgG-treated cells (Fig 3B), likely because of the effects of widespread inhibition of TrkB activity by the scavenger. TrkB inhibition in vivo, using the inhibitor ANA-12, similarly significantly reduced synapse regeneration after cooling and rewarming compared with vehicle-treated animals (Fig 3C). Thus, blocking TrkB pathway activation abolishes cooling-induced structural plasticity in wild-type neurons both in vitro and in vivo.

### RBM3 coordinates and is required for cold-induced structural plasticity

**Figure 4.**
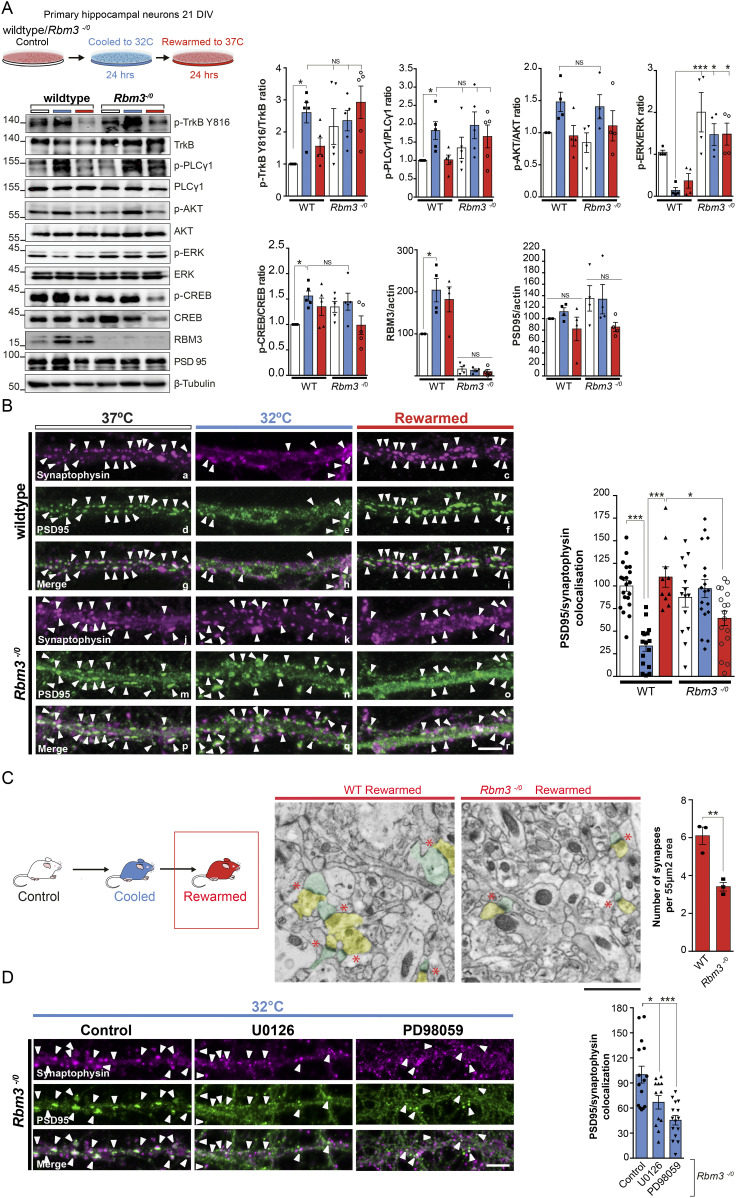
RBM3 coordinates and is required for cold-induced structural plasticity. **(A)** Schematic shows primary hippocampal neurons from *Rbm3*^−/0^ and wild-type mice cultured for 21 d and subjected to cooling at 32°C for 24 h before rewarming. Western blots show TrkB signaling on cooling in wild-type neurons as before. *Rbm3*^−/0^ neurons showed high levels of p-ERK, not low, and absent RBM3 expression. Graphs show quantification of Western blots (right). Two-way ANOVA and Tukey’s multiple comparisons test, *P < 0.05 (TrkB Tyr 816 wild-type control versus cool *P* = 0.0289; Tyr 783 PLCγ1 *P* = 0.0444; Thr202/Thr204 ERK wild-type cooled versus RBM3^−/0^ *P* = 0.0137; RBM3 wild-type control versus wild-type cooled *P* = 0.0020) and Dunnett’s multiple comparisons test (S133 CREB wild-type control versus wild-type cooled *P* = 0.0234), N.S., not significant. n = 3–5 cultures per condition. **(B)** Primary hippocampal neurons cultured from Rbm3^−/0^ mice show impaired synaptic plasticity on cooling and rewarming. Representative confocal images of control, cooled, and rewarmed neurons are shown. Neurons were immunostained with the pre-synaptic marker, synaptophysin (magenta), and the post-synaptic marker, PSD-95 (green). White arrowheads point toward synapses. Scale bar, 5 μm. (top). Bar graph shows mean normalized synapse number ± SEM. One-way ANOVA (bottom left) and Tukey’s multiple comparisons test, *P < 0.05, ***P < 0.001; N.S., not significant; (wild-type control versus wild-type cooled, *P* ≤ 0.0001, wild-type cooled versus wild-type rewarmed, *P* ≤ 0.0001; wild-type rewarmed versus *Rbm3*^−/0^ rewarmed *P* = 0.0132). n = 3 cultures per condition. **(C)** Representative electron microscopy micrographs of wild-type and *Rbm3*^−/0^ mice that have been cooled then rewarmed. The presynaptic compartment is pseudo-colored yellow and post-synaptic compartment green. Lack of RBM3 expression abrogates synapse recovery observed in wild-type mice in the CA1 region of the hippocampus. Red stars indicate synapses with pseudocoloured pre- (yellow) and post- (green) synaptic compartments. Bar chart showing mean ± SEM quantification from three animals (93 images) per condition. Two-tailed *t* test **P < 0.01 (*P* = 0.0051). Scale bar, 1 μm. n = 3 mice per condition. **(D)** ERK inhibitors restored capacity for synapse dismantling in *Rbm3*^−/0^ neurons. Representative confocal images are shown. Neurons were cooled at 32°C (24 h) and treated with vehicle (control), U0126 (10 μM, 5 h), or PD98059 (25 μM, 5 h). Neurons were immunostained with the pre-synaptic marker, synaptophysin (magenta), and the post-synaptic marker, PSD-95 (green). White arrowheads point toward synapses. Scale bar, 5 μm. Bar graph shows mean normalized synapse number ± SEM. *P < 0.05, ***P < 0.001; N.S., not significant; one-way ANOVA (right); (*P* = 0.0221 and *P* < 0.0001 and Tukey’s multiple comparisons test). n = 3 cultures per condition.

To understand to what extent cold-induced TrkB activation mediates its effects through RBM3 itself, rather than through other potential TrkB-induced effector pathways that act on synapse structure and function (Alonso et al, 2004; Minichiello, 2009; Lai et al, 2012), we tested the effects of cooling in *Rbm3*^−/0^ neurons (Matsuda et al, 2011). Levels of p-TrkB-Tyr 816 were high under all conditions of cooling and rewarming (with much greater variability than in wild-type neurons), which may reflect a role for TrkB phosphorylation in compensating for lack of RBM3. However, p-PLCγ1 and p-Akt were increased in *Rbm3*^−/0^ neurons on cooling (Fig 4A), as in wild-type neurons (Figs 4A, 1B, and 3A) but, as expected, did not induce RBM3 expression in these cells (Fig 4A). Furthermore, the suppression of p-ERK levels on cooling seen in wild-type neurons (Fig 1C) was not seen in *Rbm3*^−/0^ neurons, which instead showed sustained high levels of p-ERK (Fig 4A), consistent also with high levels of p-ERK seen when RBM3 is reduced in TrkB hemizygous Nrtk2^+/−^ mice (Fig 2B) and by ACREB expression (Fig S3A). The data provide further support that RBM3 induction exerts an inhibitory feedback loop on p-ERK activation on cooling-induced TrkB activation. Indeed, we found that Dusp6, ERK’s specific phosphatase (Pérez-Sen et al, 2019), is induced on cooling returning to normal on rewarming, a pattern that is reversed in RBM3-null neurons (Fig S3B), supporting RBM3-mediated induction of Dusp6 as a mechanism for reduction of p-ERK levels.

We next examined structural plasticity in *Rbm3*^−/0^ neurons; the effects of RBM3 knockout were twofold. First, *Rbm3*^−/0^ neurons failed to reassemble synapses after cooling–rewarming both in vitro (Fig 4B) and in vivo (Fig 4C), in contrast to wild-type neurons. This was despite activation of the upstream pathway of TrkB signaling, including increased p-PLCγ1 and p-Akt levels (Fig 4A). Second, not only was the net plasticity response defective in neurons: the whole pattern of synapse dismantling and re-assembly was altered. Thus, the number of synapses in *Rbm3*^−/0^ neurons remained unchanged during cooling compared with wild-type neurons, in which there was ∼75% reduction in synapses (*P* < 0.0001) (Fig 4B, panels b, e, h, k, n, q, and bar graph). Furthermore, rewarming did not lead to increased synapse number that characterizes structural plasticity in this paradigm (Peretti et al, 2015 and Fig 3B and C), but rather led to a significant reduction in synapses compared with wild-type neurons (Fig 4B, panels c, f, i, l, o, r, and bar graph). Thus, RBM3 expression controls synapse loss or “dismantling” during cooling as well as reassembly on rewarming. The failure to dismantle synapses was associated with high levels of p-ERK in the absence of RBM3 (Fig 4A). Treatment of *Rbm3*^−/0^ neurons with the MEK inhibitors U0126 and PD98059 (Rapoport & Ferreira, 2000; Labno et al, 2014), prevented the rise in ERK phosphorylation on cooling (Fig S3C), restoring the capacity to reduce synapse number in RBM3-null neurons (Fig 4D). Thus, low levels of p-ERK appear to be essential for the “dismantling” of synapses on cooling. Overall, we conclude that RBM3 is the key mediator of cold-induced structural plasticity as widespread TrkB activation does not compensate for loss of RBM3 function through other means.

### Pharmacological TrkB inhibition prevents cooling-induced neuroprotection in prion-diseased mice, whereas TrkB agonism induces RBM3 and is neuroprotective without cooling

Finally, we asked whether the effects of modulation of TrkB signaling on RBM3-mediated synaptic structural plasticity (Figs 3B and C and 4D) are relevant in disease. We used tg37^+/−^ mice (Mallucci et al, 2002) infected with Rocky Mountain Laboratory (RML) prions, as in our previous studies (Mallucci et al, 2003; White et al, 2008; Moreno et al, 2012; Halliday et al, 2015, 2017; Peretti et al, 2015; Bastide et al, 2017; Smith et al, 2020). Tg37^+/−^ mice express around ∼3-fold wild-type levels of prion protein (PrP) and succumb to RML prion infection 12 weeks post inoculation (w.p.i.). Synapse loss is apparent from 7 w.p.i., accompanied by cognitive deficits, followed by the onset of neuronal loss at 10 w.p.i., with the emergence of overt clinical signs and widespread neurodegeneration by 12 w.p.i. (Mallucci et al, 2002, 2003, 2007). When RML infected tg37^+/−^ mice are subjected to two cooling treatments at 3 and 4 w.p.i., early in the disease course, the neuroprotective effect is marked, preventing synapse loss and neurodegeneration, restoring memory and behavioral deficits, and significantly prolonging survival compared with non-cooled prion-diseased mice (Peretti et al, 2015). The effects are abrogated by RNAi of RBM3 and are recapitulated, in the absence of cooling, by ectopic RBM3 expression (Peretti et al, 2015) or ectopic RTN3 expression (Bastide et al, 2017).

**Figure 5.**
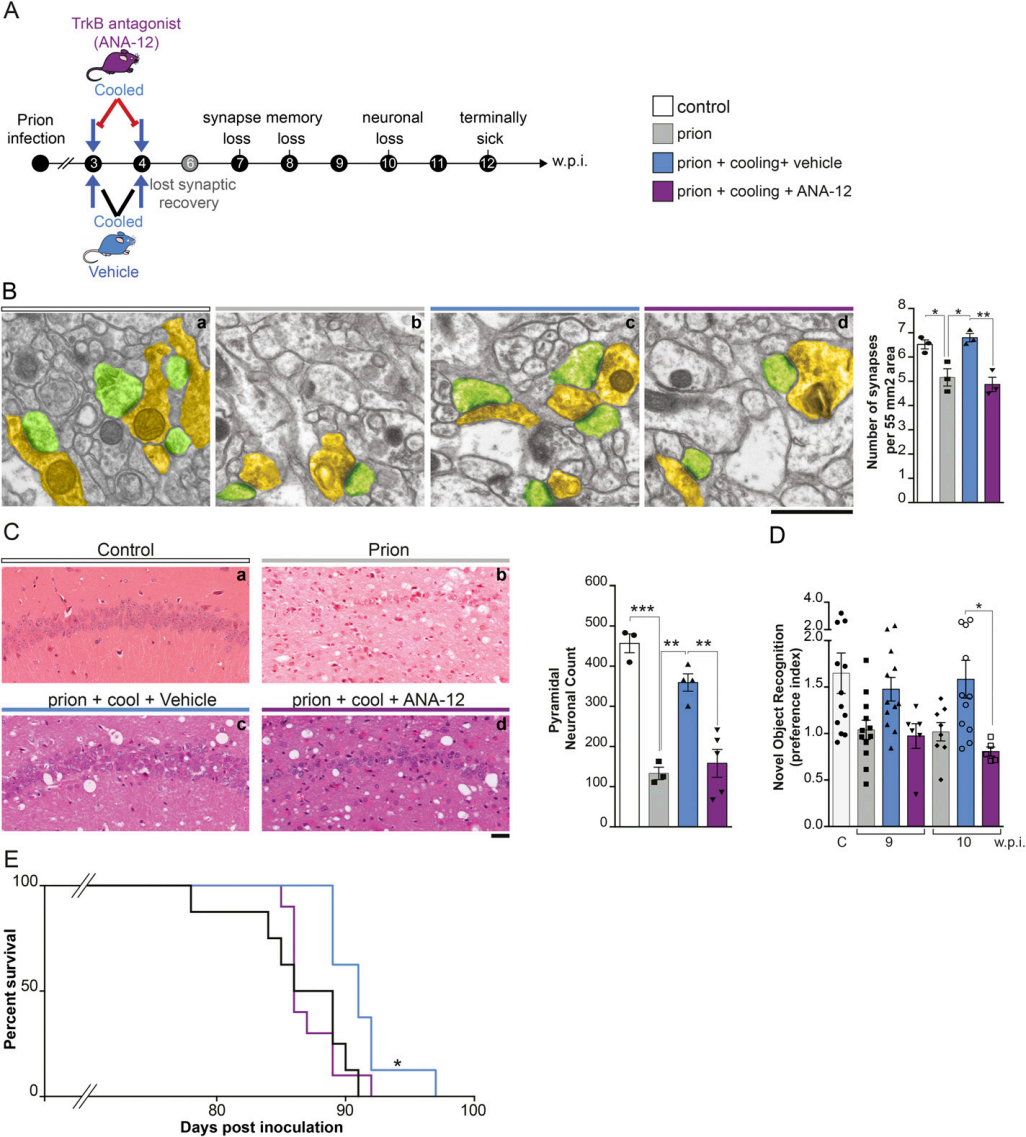
Pharmacological TrkB inhibition prevents cooling-induced neuroprotection in prion-diseased mice. **(A)** Schematic showing experimental design. Prion-diseased were treated with the non-competitive TrkB antagonist ANA-12 in conjunction with cooling at 3 and 4 w.p.i. **(B)** Representative electron microscopy micrographs showing synapses in the CA1 region of the hippocampus of prion-diseased mice at 7 w.p.i. These regenerate after cooling (panel c); which is abolished by ANA-12 (panel d). Presynaptic compartment pseudo-colored in yellow, post-synaptic compartment in green. Two-way ANOVA test and Tukey’s multiple comparison test; *P < 0.05, **P < 0.01; normal brain homogenate control versus prion (*P* = 0.0198); prion versus prion + cooled + vehicle (*P* = 0.0143); and prion + cooled + vehicle versus prion + cooled + ANA-12 (*P* = 0.0065). Bar chart shows mean ± SEM quantification from n = 3 animals (93 images) per condition. Scale bar, 1 μm. **(C)** Hematoxylin and eosin–stained sections showing the CA1 region of the hippocampus for the different experimental groups (left). ANA-12 abolished protective effects of cooling almost entirely (compare panels c and d) and see bar chart (right) showing quantification of pyramidal neuronal counts (obtained by NeuN staining). One-way ANOVA test and Tukey’s multiple comparisons test, **P < 0.01 ***P < 0.001, normal brain homogenate control versus prion (*P* = 0.0001); prion versus prion + cooled + vehicle (*P* = 0.0012) and prion + cooled + vehicle versus prion + cooled + ANA-12 (*P* = 0.0011). n = 3–5 mice per condition. Scale bar, 50 μm. **(D)** Bar chart showing novel object recognition memory test, expressed as ratio of exploratory preference for mice tested at 9 and 10 w.p.i. ANA-12 prevented the cooling induced preservation of novel object memory in diseased mice. Kruskal–Wallis test and Dunn’s multiple comparisons test, *P < 0.05; prion + cooling + vehicle 10 w.p.i. versus prion + cooling + ANA-12 10 w.p.i. (*P* = 0.0289). n = 5–12 mice per condition. **(E)** Kaplan–Meier curve showing protective effect of early cooling in prion-diseased mice (blue curve) is abolished by treatment with ANA-12 (purple line) and comparable with untreated prion-diseased mice (black line). Mann–Whitney U-test. *P < 0.05 (*P* = 0.0049), n = 9–14 mice per condition. Survival times: prion, 86.2 ± 2 d; prion + cooling + vehicle, 91.2 ± 2 d, and prion + cooling + ANA-12, 87.2 ± 2 d.

We treated RML-infected tg37^+/−^ mice with the TrkB antagonist ANA-12 or vehicle immediately before cooling at 3 and 4 w.p.i. (Fig 5A). Non-cooled RML-infected tg37^+/−^ mice and mice inoculated with normal brain homogenate were used as controls. ANA-12 treatment inhibited TrkB activation and associated RBM3 induction in hippocampi at 4 w.p.i. (Fig S3D), as in wild-type mice (Fig 2C). Consistent with our published data (Peretti et al, 2015; Bastide et al, 2017), prion-infected mice subjected to cooling showed synapse numbers in CA1 equivalent to uninfected control mice and significantly higher than in non-cooled prion-diseased mice, consistent with synapse recovery post cooling/rewarming (Fig 5B, compare panel c with panels a and b and bar graph). Similarly, cooling resulted in highly significant neuroprotection of CA1 pyramidal neurons compared with non-cooled mice (Fig 5C compare panel c with panels a and b and bar graph), prevented decline in novel object recognition memory at 9 and 10 w.p.i. that characterizes prion-diseased tg37^+/−^ mice (Mallucci et al, 2007) (Fig 5C) and significantly prolonged survival (Fig 5D). Treatment with the TrkB inhibitor, ANA-12, in contrast, abolished all the protective effects of hypothermia (Fig 5A–D). Synapse decline was equivalent to that in mice that had not been cooled and the regeneration after cooling in mice not treated with ANA-12 was prevented (Fig 5B, panels c and d and bar graph), as was pyramidal CA1 neuronal loss (Fig 5C panel d and bar graph). Furthermore, TrkB inhibitor treatment prevented the preservation of novel object recognition memory at 9 and 10 w.p.i. (Fig 5D) and, importantly, abolished the protective effects of cooling on survival (Fig 5E). Thus, preventing RBM3 induction with ANA-12 at the time of cooling abrogated neuroprotective effects in prion-diseased mice to an equivalent extent to genetic modulation by RNAi of RBM3 (Peretti et al, 2015) or RTN3 (Bastide et al, 2017).

**Figure 6.**
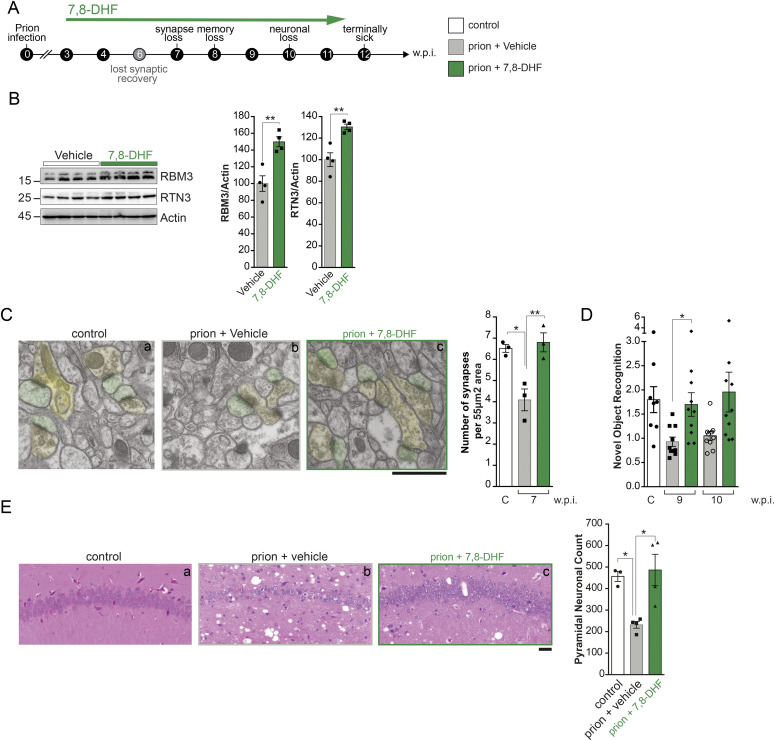
Pharmacological TrkB activation induced RBM3 without cooling and is neuroprotective in prion-diseased mice. **(A)** Schematic showing experimental design. Prion-diseased tg37 mice were treated with the TrkB agonist 7,8-DHF from 3 w.p.i. onwards. **(B)** Western blots showing sustained high levels of RBM3 and RTN3 after 7 wk of treatment at 10 w.p.i. Bar charts show mean ± SEM quantification. *t* test; **P < 0.01 RBM3, prion 10 w.p.i. + vehicle versus prion 10 w.p.i. + 7,8-DHF (*P* = 0.0075); RTN3, prion 10 w.p.i. + vehicle versus prion 10 w.p.i. + 7,8-DHF (*P* = 0.0089). n = 4 mice per condition. **(C)** Representative electron microscopy micrographs showing synapses in the CA1 region of the hippocampus of prion-diseased mice at 7 w.p.i. Presynaptic compartment pseudo-colored in yellow, post-synaptic compartment in green. 7,8-DHF rescued synapse loss in prion-diseased mice (compare panels b and c). Bar chart shows mean ± SEM quantification from n = 3 animals (93 images) per condition. Two-way ANOVA test; *P < 0.05, **P < 0.01 normal brain homogenate (NBH) control versus prion 7 w.p.i. + vehicle (*P* = 0.0134); prion 7 w.p.i. + vehicle versus prion 7 w.p.i. + 7,8-DHF (*P* = 0.008). Scale bar, 1 μm. **(D)** Novel object recognition deficits in prion-diseased mice are rescued by 7,8-DHF treatment at 9 and 10 w.p.i. Kruskal–Wallis test and Dunn’s multiple comparisons test, *P < 0.05, NBH control versus prion 9 w.p.i. (*P* = 0.0241); prion 9 w.p.i versus prion 9 w.p.i + 7,8-DHF (*P* = 0.0470); prion 9 w.p.i versus vehicle prion mice 10 w.p.i. (*P* = 0.0254). n = 8 control mice, 9 prion + vehicle mice, and 10 prion + 7,8-DHF treated mice. **(E)** Hematoxylin and eosin stained sections showing the CA1 region of the hippocampus (left). 7,8-DHF rescued pyramidal neuronal loss and reduced spongiosis in prion-diseased mice at terminal time point (12 w.p.i.) (compare panels b and c) and see bar chart (right) showing quantification of pyramidal neuronal counts (obtained by NeuN staining). One-way ANOVA test and Tukey’s multiple comparisons test, *P < 0.05 NBH control versus prion + vehicle (*P* = 0.0325; prion 9 w.p.i versus prion 9 w.p.i) + 7,8-DHF (*P* = 0.0119). Scale bar, 50 μm. n = 3–4 mice per condition.

Our data thus far predict that activation of TrkB alone, in the absence of cooling should induce the cold-shock proteins, RBM3 and RTN3, and confer neuroprotection in the context of neurodegenerative disease. We have previously confirmed that the TrkB agonist increased RBM3 expression in vivo in wild-type mice (Fig S2B). We, therefore, administered the TrkB agonist, 7,8-DHF, to prion-infected mice by intra-peritoneal injection from 3 w.p.i., the same time point at which we have previously shown cooling to be protective (Peretti et al, 2015; Bastide et al, 2017) (see schematic Fig 6A). RBM3 and RTN3 levels were significantly increased long term, with high levels seen at 10 w.p.i. (Fig 6B), with levels similar to those seen in cooling (Peretti et al, 2015). As a consequence, synapse numbers were protected (Fig 6C); memory impairment was rescued as assessed by novel object recognition memory (Fig 6D) and hippocampal pyramidal neurodegeneration was prevented (Fig 6E). This is in marked contrast to the abolition of neuroprotection in the context of cooling by TrkB inhibition in prion-infected mice (shown in Fig 5). Although other effects of TrkB activation may provide levels of protection, it is likely that these effects are, at least in part, mediated by RBM3.

## Discussion

**Figure S4.**
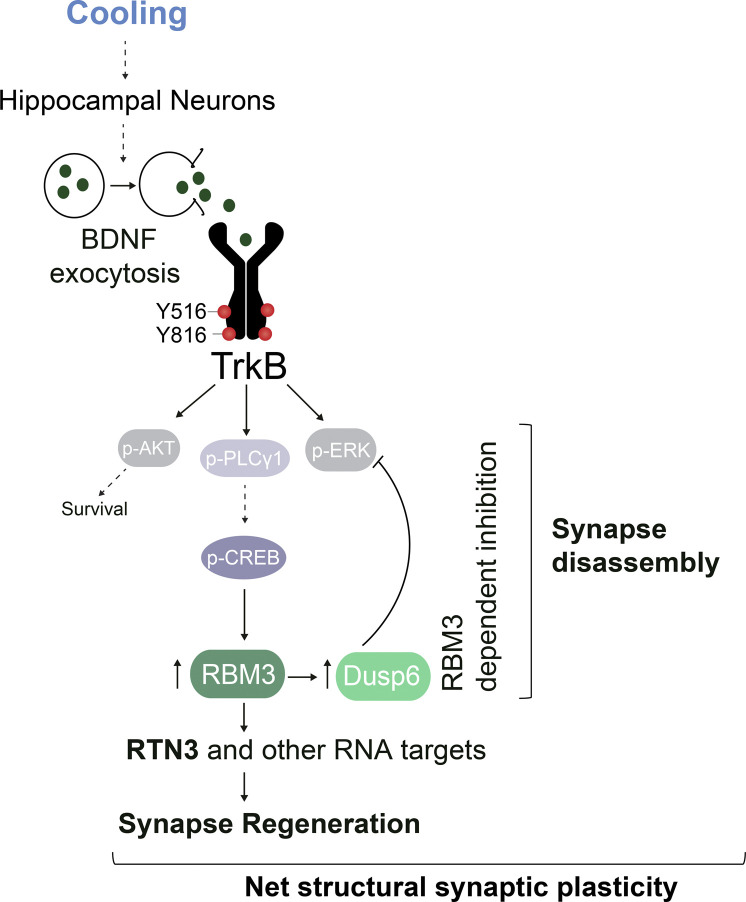
Schematic showing TrkB signaling cascade inducing RBM3 expression on cooling and mechanism by which RBM3 coordinates net structural synaptic plasticity through non-canonical feedback loop on p-ERK. Dotted arrows represent areas where additional mechanisms may contribute.

The mechanisms underlying the neuroprotective effects of cooling, a widely used therapy for brain injury, are incompletely understood. Recently, the cold-shock protein, RBM3, an RNA chaperone highly expressed in neurons, has been shown to be the principal mediator of cold-induced neuroprotection in several mouse models of neurodegenerative disease, through its effects on structural synaptic plasticity and synapse regeneration (Peretti et al, 2015). Importantly, RBM3 expression by itself is as protective as cooling. The potential to exploit this therapeutically requires elucidation of the mechanism of RBM3 expression during hypothermia, which until recently, was unknown. In this study, using the method of in vivo cooling we developed, we show that RBM3 expression is induced in parallel with TrkB receptor activation during hypothermia, with downstream activation of PLCγ1 signaling and p-CREB induction (Fig 1B) but, paradoxically, reduction of p-ERK levels (Fig 1C); see schematic in Fig S4. A similar pattern of activation has been reported in transgenic mice over-expressing TrkB receptor (Koponen et al, 2004). Importantly, genetic reduction of TrkB levels in Ntrk2^+/−^ mice (hemizygous for the TrkB receptor) abrogates this signaling, resulting in failure to induce RBM3 on cooling (Fig 2B). Furthermore, genetic interference at the level of CREB with the dominant negative CREB isoform, ACREB, blocks RBM3 induction on cooling without affecting upstream pathway activation (Fig S3A). Pharmacological inhibition of TrkB with the specific antagonist, ANA-12, also prevents cold-induced RBM3 induction and associated signaling (Fig 2C), whereas the TrkB agonist, 7,8-DHF, induces RBM3 without cooling (Fig S2B). Similarly, the BDNF scavenger, TrkB-Fc, blocks not only cold-induced TrkB-PLCγ1-CREB-RBM3 signaling in cultured neurons (Fig 3A) but also associated structural synaptic plasticity (Fig 3B), as does the small molecule TrkB inhibitor, ANA-12, in vivo (Fig 3C). TrkB-Fc also induced an increased number of synaptophysin particles during cooling and re-warming (Fig 3B), consistent with its blocking BDNF’s role in modulating the distribution of synaptic vesicles within pre-synaptic terminals (Bamji et al, 2006). Whereas cold-induced TrkB activation activates both PLCγ1- and Akt-signaling branches, ERK signaling, in contrast, is inhibited in wild-type neurons (Figs 1C, 2B, and S3). Inhibition of p-ERK activation appears to be mediated via RBM3 itself, as RBM3-deficient neurons (*Rbm3*^−/0^) show high, not low, levels of p-ERK on cooling (Figs 4A and S3). Accordingly, we show RBM3 controls the induction of Dusp6, p-ERK’s specific phosphatase cooling (Fig S3B). It would be interesting to see whether inhibition of Dusp6 and/or other Dusps is critical for ERK dephosphorylation and, conversely, whether Dups6 over-expression alters the effects of cooling-induced TrkB activation on synapse dismantling or stabilization through effects of p-ERK levels. Indeed, use of p-ERK inhibitors restored synapse dismantling capacity in RBM3-null neurons on cooling, consistent with this (Fig 4D). p-ERK inhibition in association with high RBM3 levels has been reported in response to rotenone-mediated activation of multiple MAPK-associated signaling pathways in a neuronal cell line, but not in association with TrkB activation (Yang et al, 2019). In contrast, in rats treated with mild hypothermia for sub-arachnoid hemorrhage, p-TrkB/p-ERK/p-CREB levels all increased on cooling, but significant levels of p-ERK preceded activation of TrkB receptor and RBM3 levels were not measured (Lv et al, 2016). Our data support the reduction of p-ERK levels to be necessary for synapse dismantling on cooling as loss of inhibition of ERK activation in *Rbm3*^−/0^ neurons results in the failure of these cells to disassemble synapses during hypothermia before their regeneration on re-warming (Fig 4B and C). The use of ERK inhibitors in *Rbm3*^−/0^ neurons restores this capacity, as discussed (Fig 4D). Increased dendritic spine density in hippocampal CA1 neurons (Alonso et al, 2004) and increased PSD-95 levels in synapses (Yoshii & Constantine-Paton, 2014) involve BDNF-TrkB-p-ERK signaling (see Smolen et al [2019] for review), consistent with low levels of p-ERK being associated with synapse reduction here. ERK inhibitors could affect synapse number in both wild-type and *Rbm3*^−/0^ neurons under control conditions through actions on canonical TrkB-ERK actions. However, pERK levels are low in the absence of agonist under baseline conditions (Fig S3C), so effects of inhibitors are likely to be minimal. The explanation for the apparent paradox of the same pathway controlling the dismantling synapses during cooling and their reassembly on rewarming condition may lie in the timeframes of the dynamics of the two phenomena we observed in this study: the phosphorylation/dephosphorylation dynamics that usually occur within seconds/minutes; and the synthesis rates of new proteins, including RBM3, which has a regulatory hub role, or its downstream targets Dusp6 and RTN3 (and potentially many others) within a timeframe of minutes/hours. How this is coordinated would be very interesting to understand but may be regulated, at least in part, by the recently described effects of RBM3 on hippocampal neuronal activity through local synaptic translation (Sertel et al, 2020).

The pathway is also of key importance in mediating structural plasticity in neurodegenerative diseases. In prion-diseased mice, use of the TrkB inhibitor ANA-12 at the time of cooling abrogated all neuroprotective effects, including synapse regeneration, prevention of neuronal loss and cognitive deficits, and prolonged survival (Fig 5). Inhibiting TrkB activation is thus as potent a blocker of cold-induced RBM3-mediated neuroprotection as genetic knockdown of RBM3 expression. In contrast, use of the TrkB agonist 7,8-DHF increased RBM3 levels without cooling in prion-diseased mice with associated neuroprotective effects (Fig 6). Clearly, other TrkB-mediated processes could be implicated in this protective effect, but we propose RBM3 expression is at least partly responsible given the effects of TrkB inhibition seen in Fig 5. Importantly, we found that widespread TrkB activation did not compensate for lack of RBM3 in both *Rbm3*^−/0^ cultured neurons and in RBM3-null mice, which lacked structural synaptic plasticity on cooling (Fig 4B and C). The data thus support targeting the pathway pharmacologically at various levels—from TrkB downwards—for the induction of RBM3. Of course, TrkB activators including 7,8-DHF and related molecules (Jang et al, 2010; Chen et al, 2018) are well known to have neuroprotective effects on synapses, improving learning and memory and reducing neuronal loss in various disease models (Devi & Ohno, 2012; Wu et al, 2014; Zhang et al, 2014; Barriga et al, 2017; Nie et al, 2019), which may, at least in part be due to RBM3 induction. A number of anti-depressants are known to induce TrkB activation (Björkholm & Monteggia, 2016), and it will be interesting to test their capacity to induce RBM3 through this pathway. Critically, the ability to induce RBM3 downstream of the receptor is important, as not only BDNF, but also TrkB receptor levels are known to decrease over time in the brains of Alzheimer’s patients (Allen et al, 1999; Tapia-Arancibia et al, 2008). Interestingly, mutation of the Y816 site, but not Y515, of the TrkB receptor has been found a major effect on synaptic plasticity (Minichiello, 2002).

Many aspects of the pathway remain unknown, including how cooling induces BDNF release. The downstream RNA targets of RBM3, other than RTN3, also remain unknown, as does the precise mechanism by which synapses are disassembled and regenerated also and are all questions for future studies. Possible mechanisms contributing to synapse regeneration relate to the dendritic localization of the specific RBM3 isoform that rescues synapse loss in disease (Peretti et al, 2015), which is associated with increased global protein synthesis rates (Smart et al, 2007; Jackson et al, 2015), but also, most recently has been shown to control neuronal endogenous activity through local translation at synapses (Sertel et al, 2020). Furthermore, high expression of RBM3 induces the formation of long protrusions with F-actin component in several cell types (Pilotte et al, 2018), and its reduction compromises neuronal differentiation and cell polarity and migration (Pilotte et al, 2018; Xia et al, 2018). Whether these processes are regulated by the TrkB—pathway we describe—and the role of p-ERK in these phenomena will be interesting to test.

Collectively, our findings show that RBM3 induction on cooling occurs through and results in a non-canonical pattern of TrkB activation, through which structural plasticity is coordinated. This is abolished in RBM3-null neurons, confirming RBM3 as a major mediator of the response even with widespread TrkB activation. We show this signaling can be exploited for induction of RBM3 and its neuroprotective effects without cooling, whereas blocking it abrogates the protective effects of hypothermia. Both genetic and pharmacological data confirm that TrkB signaling is both necessary and sufficient for RBM3 induction and its downstream protective effects, which can now be exploited therapeutically without the need for cooling.

## Materials and Methods

### Primary neuronal culture

Primary neurons were isolated from the hippocampi of both male and female C57Bl6/N and *Rbm3*^−/0^ (http://www.informatics.jax.org/allele/MGI:5285686) mouse pups at postnatal day 0 or 1. For the isolation and culture of hippocampal neurons, we followed the protocol of Beaudoin et al (2012) with slight modifications (Beaudoin et al, 2012). Briefly, hippocampi were extracted into Hibernate A media (Gibco) and incubated at 37°C with papain solution for 20 min. Papain solution was removed and trypsin inhibitor was added for 5 min. Hippocampi were then washed three times in prewarmed plating media (Neurobasal A, B27 supplement, GlutaMAX, Horse serum, 1M Hepes, pH 7.5) before being triturated 8–10 times. The suspension was strained and 300,000 cells were seeded onto glass coverslips coated with poly-L-lysine. Media were changed to neuron media (Neurobasal A, B27 supplement, GlutaMAX, Penicillin/Streptomycin) 4 h post-seeding. Primary neurons were maintained at 37°C, 5% CO_2_ or as indicated at 32°C. 5-fluoro-2′-deoxyuridine (Sigma-Aldrich) was added at a final concentration of 7.15 μg/ml to inhibit glial growth (Vossel et al, 2015). A third of the media was changed for fresh media every 4–5 d. Neurons were plated for Western blot in 6-well plate at 72,300 cells/cm^2^ and for immunofluorescence analysis on 13 mm coverslips (Hecht-assistant) at 31,200 cells/cm^2^. Experimental procedures lasting 24–48 h were started at day 19–20 D.I.V. to finish at 21 D.I.V. (days in vitro). Tetanus neurotoxin (R&D Systems, Bio-Techne) was used at a final concentration of 10 nM. TrkB-Fc was used at a final concentration of 5 μg/ml in the media (Cheng et al, 2011). ERK kinases inhibitors U0126 and PD98059 were dissolved as recommended (Tocris Biosciences) and used at a final concentration of 10 and 25 μM, respectively.

### Immunocytochemistry for in vitro synapse quantification

Neurons were fixed in a 4% paraformaldehyde, 4% sucrose solution for 20 min. Coverslips were washed with PBS then incubated with 0.1% Triton X-100 for 5 min, followed by 50 mM NH_4_Cl for 20 min. Neurons were then blocked in 10% goat serum for 1 h at room temperature. Synaptophysin (1:1,000; catalog number: 101002; Synaptic Systems) and PSD-95 (1:200; catalog number: MABN68; Millipore) antibodies were diluted in blocking solution and were incubated overnight at 4°C. Following washes in PBS, neurons were incubated for 1 h at room temperature with anti-mouse Alexa Fluor 488 and antirabbit Alexa Fluor 568 (1:1,000; Thermo Fisher Scientific). Coverslips were stained with DAPI before mounting. Images were captured using a Leica confocal microscope at 63× magnification. The number of co-localized puncta was quantified using the JACoP plugin in ImageJ.

### Mice

All animal work conformed to UK Home Office regulations and performed under the Animal (Scientific Procedures) Act 1986, Amendment Regulations 2012 and following institutional guidelines for the care and use of animals for research. All studies were ethically reviewed by the University of Cambridge Animal Welfare and Ethical Review Body (AWERB). Mice were housed in groups of 2–5 animals/cage, under 12 h light/dark cycle, and were tested in the light phase. Water and standard mouse chow were given ad-libitum. Mice were randomly assigned treatment groups by cage number. Experimenters were blind to group allocation during the experiments and when assessing clinical signs. For behavioral testing no formal randomization was needed or used. Procedures were fully compliant with Animal Research: Reporting of In Vivo Experiments (ARRIVE) guidelines.

### Induction of hypothermia

C57Bl6/J wild-type mice, tg37^+/−^ (Mallucci et al, 2002) and B6;129S2-Ntrk2^tm1Bbd^/J (Ntrk2^+/−^) mice (https://www.jax.org/strain/002544) weighing ≥20 g were cooled using 5′-AMP as described (Peretti et al, 2015). Briefly, mice were intraperitoneally injected with freshly prepared 5′-AMP (0.7 mg per g; Sigma-Aldrich) or saline control. Mice were maintained at room temperature until core body temperature decreased to 25°C (∼60 min). Subsequently, mice were transferred to a refrigerator (5–10°C) and core body temperature lowered to 16–18°C for 45 min. For rewarming, mice were allowed to recover to normal body temperature at room temperature conditions. Cooled samples were collected at the end of the 16–18°C period and rewarmed samples as stated elsewhere in the text. ANA-12 (N-[2-[[(Hexahydro-2-oxo-1H-azepin-3- yl)amino]carbonyl]phenyl]benzo[b]thiophene-2-carboxamide) (Tocris) compound was intraperitoneally injected (0.5 mg/kg in 0.5% DMSO in saline solution) in wild-type and prion-infected mice, twice a day for 2 d before cooling and 2 h before the initiation of the cooling protocol. 7,8-Dihydroxyflavone (7,8-DHF) (Tocris Bioscience) was injected intraperitoneally to C57Bl6/J wild-type mice at 5 mg/kg in 17% DMSO in PBS buffer (Devi & Ohno, 2012).

### Prion infection of mice

3-wk-old tg37^+/−^ mice were inoculated intra-cerebrally into the right parietal lobe with 30 μl of 1% brain homogenate of Chandler/RML (Rocky Mountain Laboratories) prions under general anesthetic, as described (Mallucci et al, 2002). Animals were culled when they developed clinical signs of scrapie as defined in (Mallucci et al, 2002, 2003, 2007). Control mice received 1% normal brain homogenate. Administration of the small specific TrkB agonist 7,8-DHF to prion mice from 3 w.p.i. was performed with a series of daily doses via intraperitoneal administration (5 mg/kg) for 10 consecutive days, followed by 3-d interval. This protocol was repeated until mice were terminally ill.

### AAVs

We generated AAV5-CamkIIa-GFP-T2A-ACREB from an Addgene vector (Lubelski et al, 2012) containing GFP-T2A-ACREB. The neuron-specific promoter CAMKIIa was used as previously (Peretti et al, 2015) AAVs were injected stereotaxically into the CA1 region of the hippocampus as described (White et al, 2008). Vector BioLabs generated the viral particles with a final titer of 1.3 × 10^13^ GC/ml for control virus and 4.9 × 10^12^ GC/ml for ACREB virus. Viruses were injected stereotaxically into the CA1 region of the hippocampus as described (White et al, 2008). Samples were collected 2 wk after viral injection.

### Electron microscopy data acquisition and analysis of synapse number

Male mice were used to avoid the effects of the estrus cycle on synapse number (Peretti et al, 2015). Brains were perfusion fixed with 2% glutaraldehyde and 2% paraformaldehyde in 0.1 M sodium cacodylate buffer (final pH 7.3). Slices (300 mm) were prepared using a vibrating blade microtome (Leica Microsystems). These slices were post-fixed in 1% osmium tetroxide, 11% potassium ferrocyanide, stained en-bloc with 5% uranyl acetate and embedded in epoxy resin (TAAB Laboratories Equipment Ltd), as described (https://www.gatan.com/techniques/serial-block-face-imaging). For routine 2D analyses, semi-thin (1 mm) sections were stained with toluidine blue and examined to select areas for ultramicrotomy. Ultrathin sections (∼70 nm) were stained with lead citrate and examined, blind, in a Jeol 100-CXII electron microscope (JEOL(UK) Ltd) equipped with a “Megaview III” digital camera (Olympus Soft Imaging Solutions GmbH) for Fig 3C. For Fig 4C, ultrathin sections (∼80 nm) were cut using a Leica Ultracut ultramicrotome and mounted on melinex plastic coverslips. Coverslips are mounted on aluminum SEM stubs using carbon sticky pads and coated with 30 nm carbon. Samples are imaged in an FEI Verios 460 SEM at 3–4 keV accelerating voltage, 0.2 nA probe current in backscatter mode using the concentric backscatter detector at a dwell time of 3 µs. Image maps were acquired using FEI MAPS software (12.7 μm tile size, i.e., 10,000 magnification, 1,536 × 1,024 pixel resolution). Images were recorded from the stratum radiatum, all at a distance of ∼100 mm from the CA1 pyramidal layer to avoid the large dendritic profiles in the proximal area. 31 images, each encompassing an area of 55 mm^2^, from each of two to three mice were used for scoring. For synapse quantification the following criteria were followed: the presence of an unambiguous postsynaptic density, a clear synaptic cleft, and three or more synaptic vesicles. An average of 300 synapses were counted per sample. Quantification of numbers of neurons was performed in the CA1 region of hippocampus.

### Novel object recognition memory testing

This was performed as described (Mallucci et al, 2007). Briefly, mice were tested in a black cylindrical arena (69 cm diameter) mounted with a 100 LED strip infrared light source and a high-resolution day/night video camera (Sony). Mice were acclimatized to the arena 5 d before testing. During the learning phase, two identical objects were placed 15 cm from the sides of the arena. Each mouse was placed in the arena by an operator blind to the experimental group for two blocks of 10 min for exploration of the objects with an inter-trial interval of 10 min. 2 h later, one of the objects was exchanged for a novel one, and the mouse was replaced in the arena for 5 min (test phase). The amount of time spent exploring all objects was tracked and measured for each animal using Ethovision software (Tracksys). All objects and the arena were cleaned thoroughly between trials to ensure the absence of olfactory cues. The amount of time spent exploring the novel object over the familiar object is expressed as a ratio, where a ratio of 1 reflects random exploration, and >1 reflects memory. Behavioral data were analyzed using one-way ANOVA with Brown–Forsythe test and Tukey’s post hoc test. For behavioral testing no formal randomization was needed or used. Experimenter was blind to group allocation during all experiments and when assessing outcome.

### Histology

Paraffin-embedded brains were sectioned at 5 mm and stained with H&E as described (Moreno et al, 2012). Neuronal counts were determined by quantifying NeuN-positive pyramidal CA1 neurons as described (Moreno et al, 2012).

### Immunoblotting

Protein samples were isolated from hippocampi using protein lysis buffer (50 mM Tris, 150 mM NaCl, 1% Triton X-100, 1% Na deoxycholate, 0.1% SDS, and 125 mM sucrose) supplemented with Phos-STOP and protease inhibitors (Complete; Roche), followed by centrifugation and quantification. Protein levels were determined by resolving 10–20 μg of protein on SDS–polyacrylamide gels, transferred onto either nitrocellulose or PDVF membranes and incubated with primary antibodies. Proteins were detected using the following antibodies: TrkB, p-TrkB γ816, and p-TrkB γ516 (1:1,000; catalog number: 4603, 4168, 4619; Cell Signaling Technology); PLCγ1 and p-PLCγ1 γ783 (1:1,000; catalog number: 2822 and 2821; Cell Signaling Technology); AKT and p-AKT Ser473 (1:1,000; catalog number: 9272, 9271; Cell Signaling Technology), p44/42 MAPK (Erk1/2) and p-p44/42 MAPK (p-Erk1/2) (1:1,000; catalog number: 4695, 4376; Cell Signaling Technology); CREB and p-CREB Ser133 (1:1,000; catalog number: 9104, 9198; Cell Signaling Technology); RBM3 (1:1,000; catalog number: 14363-1-AP; ProteinTech); RTN3 (custom made in rabbit against RTN3 peptide 213–227 a.a. H-CARDQTKSIVEKIQAK-NH2 [Sigma-Aldrich] by UKSBS); MKP3/Dusp6 (1:1,000; catalog number: sc-377070; Santa Cruz Technology), and PSD95 (1:1,000; catalog number: 04-1066; Millipore). Horseradish peroxidase–conjugated secondary antibodies (1:10,000; DAKO) were applied and protein visualized using enhanced chemiluminescence (GE Healthcare) and quantified using ImageJ. GAPDH (1:5,000; catalog number: sc32233; Santa Cruz Technology), actin (1:5,000; catalog number MAB1501R; Millipore), or β-tubulin (ab179513; Abcam) were used as loading controls depending on molecular weight or availability.

### Statistical analysis

Data are presented as the mean ± SEM unless otherwise specified in the legend. Statistical significance was determined using GraphPad Prism v8, using a *t* test or one-way ANOVA with Tukey’s test for multiple comparisons for normally distributed data or Kruskal–Wallis test and Dunn’s multiple comparisons test for non-normally distributed data sets. Statistical significance was accepted at *P* ≤ 0.05. In the figure legends, “ns”*P* ≥ 0.05, *P ≤ 0.05, **P ≤ 0.01 and ***P ≤ 0.001.

## Supplementary Material

Reviewer comments
