# Supplementary figures and images for: TrkB signaling regulates the cold-shock protein RBM3-mediated neuroprotection
Source: Life Sci Alliance. 2021 Feb 9;4(4):e202000884. doi: 10.26508/lsa.202000884 (PMC7893816; doi:10.26508/lsa.202000884)

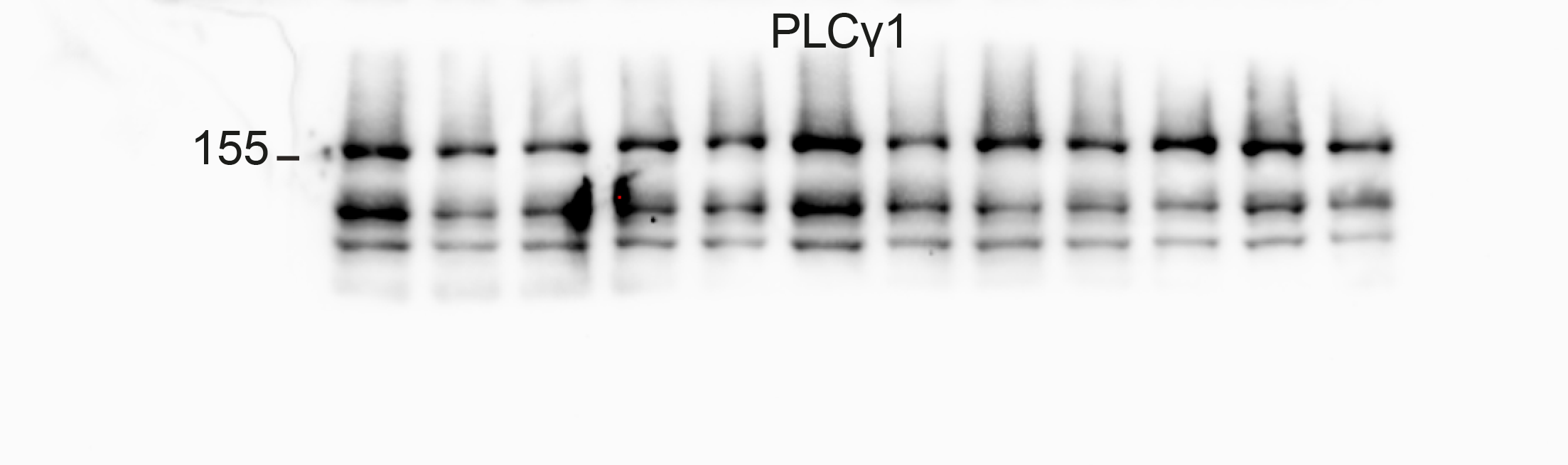

Supplement: Supplementary file 1 [file LSA-2020-00884_SdataF2.tif]

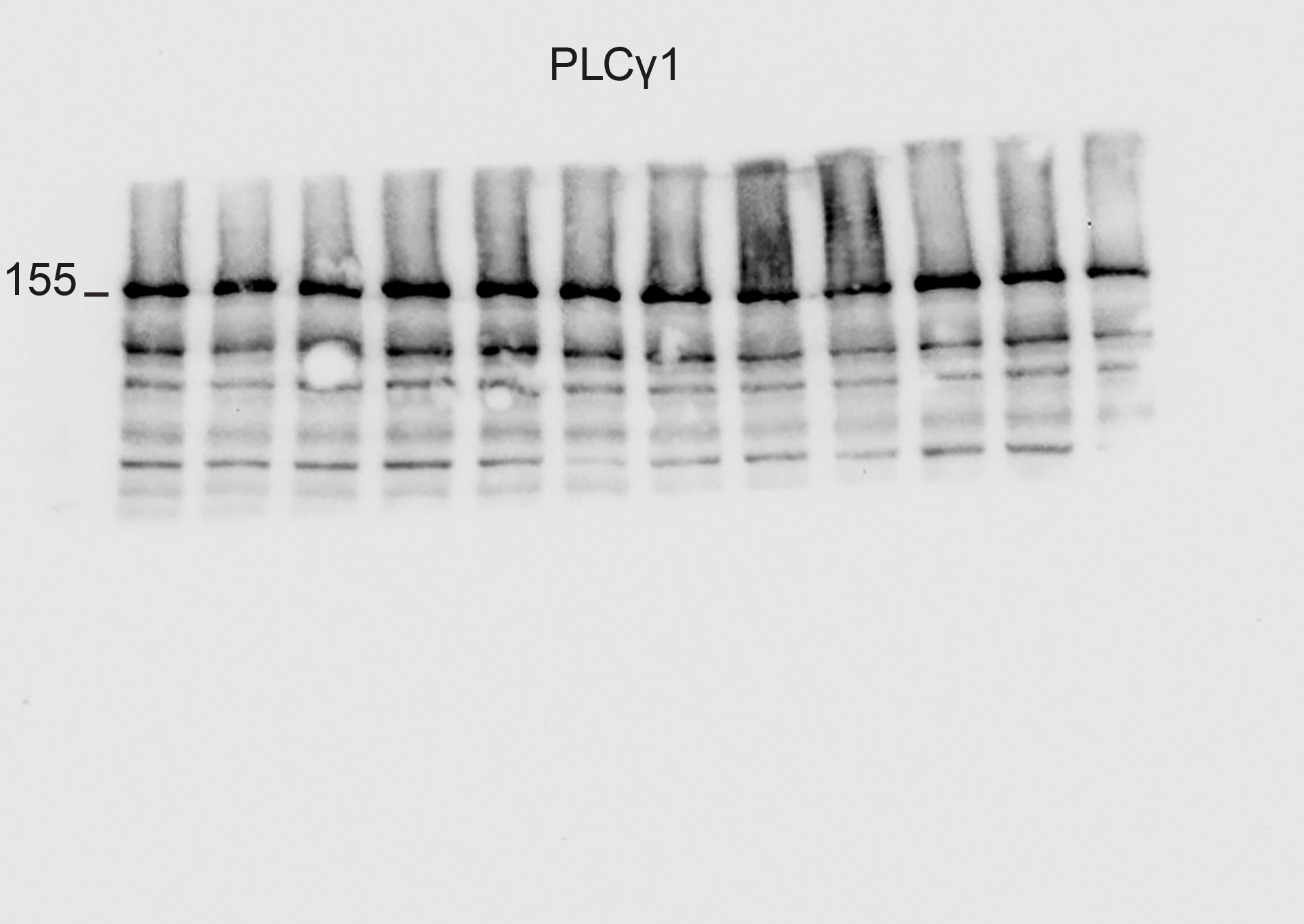

Supplement: Supplementary file 2 [file LSA-2020-00884_SdataFS3.tif]
