# Supplementary material for: TrkB signaling regulates the cold-shock protein RBM3-mediated neuroprotection
Source: Life Sci Alliance. 2021 Feb 9;4(4):e202000884. doi: 10.26508/lsa.202000884 (PMC7893816; doi:10.26508/lsa.202000884)
Supplement: Reviewer comments [file LSA-2020-00884_review_history.pdf]

Life Science Alliance

TrkB signaling regulates the cold-shock protein RBM3-mediated neuroprotection

Diego Peretti, Heather Smith, Nicholas Verity, Ibrahim Humoud, Lis de Weerd, Dean Swinden, Joseph Hayes, and Giovanna Mallucci
DOI: https://doi.org/10.26508/lsa.202000884

Corresponding author(s): Giovanna Mallucci, University of Cambridge

Review Timeline:

Submission Date:	2020-08-22
Editorial Decision:	2020-09-25
Revision Received:	2020-12-20
Editorial Decision:	2021-01-20
Revision Received:	2021-01-25
Accepted:	2021-01-25

Scientific Editor: Shachi Bhatt

Transaction Report:

(Note: With the exception of the correction of typographical or spelling errors that could be a source of ambiguity, letters and reports are not edited. The original formatting of letters and referee reports may not be reflected in this compilation.)

No full Peer Review Process File is available with this article, as the authors have chosen not to make the review process public in this case.

September 25, 2020

Re: Life Science Alliance manuscript #LSA-2020-00884-T

Prof. Giovanna rachele mallucci
University of Cambridge
UK DRI at University of Cambridge
Island Research Building
Cambridge cb2 0ah
United Kingdom

Dear Dr. Mallucci,

Thank you for submitting your manuscript entitled "TrkB signaling regulates RBM3-mediated neuroprotection" to Life Science Alliance (LSA). The manuscript has been reviewed by the editors and outside referees (reviewer comments below). As you will see, the reviewers were enthusiastic about the study and its potential impact, but have raised a number of concerns that should be addressed prior to further consideration of the manuscript at LSA. Therefore, although we are unable to publish the current version of the manuscript, we would encourage you to submit a revised version that addresses the reviewers' concerns. While we appreciate the detailed review by referee # 3, figuring out the downstream effects of RBM3 on DUSP6, Erk and BDNF signaling, and synapse dismantling (R3 pt 1, 2) and experiments showing the effects of antidepressants that induce RBM3 expression (R3 pt5) should only be included in the revised manuscript if readily attainable. Otherwise, a discussion of these points in the revised manuscript will suffice.

We would be happy to discuss the individual revision points further with you, should this be helpful. A revised manuscript may be re-reviewed, most likely by some or all of the original referees. When submitting the revision, please include a letter addressing the reviewers' comments point-by-point and a copy of the text with alterations highlighted (boldfaced or underlined). The typical time frame for revisions is three months. In an effort to expedite the review process, papers are generally considered through only one revision cycle.

While you are revising your manuscript, please also attend to the below editorial points to help expedite the publication of your manuscript.

To upload the revised version of your manuscript, please log in to your account:
https://lsa.msubmit.net/cgi-bin/main.plex
You will be guided to complete the submission of your revised manuscript and to fill in all necessary information. Please get in touch in case you do not know or remember your login name.

Thank you for this interesting contribution to Life Science Alliance. We are looking forward to receiving your revised manuscript.

Sincerely,

Shachi Bhatt, Ph.D.
Executive Editor

Life Science Alliance

A. THESE ITEMS ARE REQUIRED FOR REVISIONS

-- A letter addressing the reviewers' comments point by point.

-- An editable version of the final text (.DOC or .DOCX) is needed for copyediting (no PDFs).

-- High-resolution figure, supplementary figure and video files uploaded as individual files: See our detailed guidelines for preparing your production-ready images, https://www.life-science-alliance.org/authors

-- Summary blurb (enter in submission system): A short text summarizing in a single sentence the study (max. 200 characters including spaces). This text is used in conjunction with the titles of papers, hence should be informative and complementary to the title and running title. It should describe the context and significance of the findings for a general readership; it should be written in the present tense and refer to the work in the third person. Author names should not be mentioned.

B. MANUSCRIPT ORGANIZATION AND FORMATTING:

Full guidelines are available on our Instructions for Authors page, https://www.life-science-alliance.org/authors

We encourage our authors to provide original source data, particularly uncropped/-processed electrophoretic blots and spreadsheets for the main figures of the manuscript. If you would like to add source data, we would welcome one PDF/Excel-file per figure for this information. These files will be linked online as supplementary "Source Data" files.

IMPORTANT: It is Life Science Alliance policy that if requested, original data images must be made available. Failure to provide original images upon request will result in unavoidable delays in publication. Please ensure that you have access to all original microscopy and blot data images before submitting your revision.

January 20, 2021

RE: Life Science Alliance Manuscript #LSA-2020-00884-TR

Prof. Giovanna rachele mallucci
University of Cambridge
UK DRI at University of Cambridge
Island Research Building
Cambridge cb2 0ah
United Kingdom

Dear Dr. Mallucci,

Thank you for submitting your revised manuscript entitled "TrkB signaling regulates the cold-shock protein RBM3-mediated neuroprotection". We would be happy to publish your paper in Life Science Alliance pending final revisions necessary to meet some of reviewers' concerns and our formatting guidelines.

As you will note from the reviewers' comments below, while Rev 1 and 3 are satisfied with the revised manuscript, Rev 2 still has some reservations. We are sympathetic to your situation, in that it would take an unreasonable amount of time to collect data to provide the controls that Rev 2 had requested. In lieu of that data, we do suggest that you explicitly state the caveats of these experiments (given that the controls requested are not included) and cautiously discuss the interpretations. All other minor edits requested by Rev 1 and 3 should also be addressed. We encourage you to include a pbp rebuttal to the reviewers' points.

Along with the points listed below, please also attend to the following:
-please make sure the author order in your manuscript and our system match
-please upload your main and supplementary figures as single files
-please add callouts for Figure S2 A and B to your main manuscript text
-please use the [10 author names, et al.] format in your references (i.e. limit the author names to the first 10)
-please provide higher resolution images for all the western blots (Fig 1B, C, 2B, C, 3A, 4A, 6B, S1A, B, S2B, S3A-D) in the manuscript
-please provide source data (unedited uncropped original images) for the PLCy1 blots in Fig 2B AND S3A

If you are planning a press release on your work, please inform us immediately to allow informing our production team and scheduling a release date.

To upload the final version of your manuscript, please log in to your account:
https://lsa.msubmit.net/cgi-bin/main.plex
You will be guided to complete the submission of your revised manuscript and to fill in all necessary information. Please get in touch in case you do not know or remember your login name.

To avoid unnecessary delays in the acceptance and publication of your paper, please read the following information carefully.

A. FINAL FILES:

These items are required for acceptance.

-- An editable version of the final text (.DOC or .DOCX) is needed for copyediting (no PDFs).

-- High-resolution figure, supplementary figure and video files uploaded as individual files: See our detailed guidelines for preparing your production-ready images, https://www.life-science-alliance.org/authors

-- Summary blurb (enter in submission system): A short text summarizing in a single sentence the study (max. 200 characters including spaces). This text is used in conjunction with the titles of papers, hence should be informative and complementary to the title. It should describe the context and significance of the findings for a general readership; it should be written in the present tense and refer to the work in the third person. Author names should not be mentioned.

B. MANUSCRIPT ORGANIZATION AND FORMATTING:

Full guidelines are available on our Instructions for Authors page, https://www.life-science-alliance.org/authors

We encourage our authors to provide original source data, particularly uncropped/-processed electrophoretic blots and spreadsheets for the main figures of the manuscript. If you would like to add source data, we would welcome one PDF/Excel-file per figure for this information. These files will be linked online as supplementary "Source Data" files.

Submission of a paper that does not conform to Life Science Alliance guidelines will delay the acceptance of your manuscript.

It is Life Science Alliance policy that if requested, original data images must be made available to the editors. Failure to provide original images upon request will result in unavoidable delays in publication. Please ensure that you have access to all original data images prior to final submission.

The license to publish form must be signed before your manuscript can be sent to production. A link to the electronic license to publish form will be sent to the corresponding author only. Please take a moment to check your funder requirements.

Reviews, decision letters, and point-by-point responses associated with peer-review at Life Science Alliance will be published online, alongside the manuscript. If you do want to opt out of having the reviewer reports and your point-by-point responses displayed, please let us know immediately.

Thank you for your attention to these final processing requirements. Please revise and format the manuscript and upload materials within 7 days.

Thank you for this interesting contribution, we look forward to publishing your paper in Life Science Alliance.

Sincerely,

Shachi Bhatt, Ph.D.
Executive Editor
Life Science Alliance
https://www.lsajournal.org/
Tweet @SciBhatt @LSAjournal

January 25, 2021

RE: Life Science Alliance Manuscript #LSA-2020-00884-TRR

Prof. Giovanna Rachele Mallucci
University of Cambridge
UK DRI at University of Cambridge
Island Research Building
Cambridge cb2 0ah
United Kingdom

Dear Dr. Mallucci,

Thank you for submitting your Research Article entitled "TrkB signaling regulates the cold-shock protein RBM3-mediated neuroprotection". It is a pleasure to let you know that your manuscript is now accepted for publication in Life Science Alliance. Congratulations on this interesting work.

The final published version of your manuscript will be deposited by us to PubMed Central upon online publication.

Your manuscript will now progress through copyediting and proofing. It is journal policy that authors provide original data upon request.

Reviews, decision letters, and point-by-point responses associated with peer-review at Life Science Alliance will be published online, alongside the manuscript. If you do want to opt out of having the reviewer reports and your point-by-point responses displayed, please let us know immediately.

IMPORTANT: If you will be unreachable at any time, please provide us with the email address of an alternate author. Failure to respond to routine queries may lead to unavoidable delays in publication.

Scheduling details will be available from our production department. You will receive proofs shortly before the publication date. Only essential corrections can be made at the proof stage so if there are any minor final changes you wish to make to the manuscript, please let the journal office know now.

DISTRIBUTION OF MATERIALS:
Authors are required to distribute freely any materials used in experiments published in Life Science Alliance. Authors are encouraged to deposit materials used in their studies to the appropriate repositories for distribution to researchers.

You can contact the journal office with any questions, contact@life-science-alliance.org

Again, congratulations on a very nice paper. I hope you found the review process to be constructive and are pleased with how the manuscript was handled editorially. We look forward to future exciting submissions from your lab.

Sincerely,

Shachi Bhatt, Ph.D.
Executive Editor
Life Science Alliance
https://www.lsajournal.org/
Tweet @SciBhatt @LSAjournal
